# Integrating CRISPR genome editing with liver organoid and hiPSC-derived microfluidic platforms to model metabolic dysfunction–associated steatotic liver disease

**DOI:** 10.1016/j.bbrep.2026.102616

**Published:** 2026-05-19

**Authors:** Aina Kehinde Oluwasegun

**Affiliations:** Friedrich Schiller University Jena, Germany

**Keywords:** MASLD, CRISPR, Organoids, hiPSCs

## Abstract

Metabolic dysfunction–associated steatotic liver disease (MASLD) and its progressive form, metabolic dysfunction–associated steatohepatitis (MASH), arise from the interplay of genetic susceptibility, metabolic stress, and multicellular hepatic dysfunction. However, causal interpretation of identified risk and protective variants remains constrained by the lack of human-relevant model systems.

This review examines how dissecting the pathophysiological roles of MASLD-associated variants can refine our understanding of disease mechanisms and inform in vitro modeling strategies. It focuses on how human hepatic systems derived from induced pluripotent stem cells (hiPSCs), integrated with advances in Interspaced CRISPR-based genome editing, can aid our understanding of enabling precise genetic manipulation and the generation of isogenic, Human Leukocyte Antigen (HLA) -matched models for liver research.

Lastly, by combining these approaches with liver differentiation protocols, organoid systems, and liver-on-chip platforms, a robust framework emerges for modeling patient-specific genetic variation. This integrated strategy will support mechanistic studies, therapeutic target validation, and the evaluation of safety and efficacy for genome-editing interventions in MASLD.

## Highlights

• CRISPR-edited hiPSC liver models allow isogenic study of MASLD/MASH risk variants.
• Liver organoids and microphysiological systems recreate multicellular tissue crosstalk.
• Genome editing + liver-on-chip bridges gaps between in vitro models and human disease.
• LoCs and organoids speed translation in drug development and clinical research.

## Graphical abstract

**Figure:**
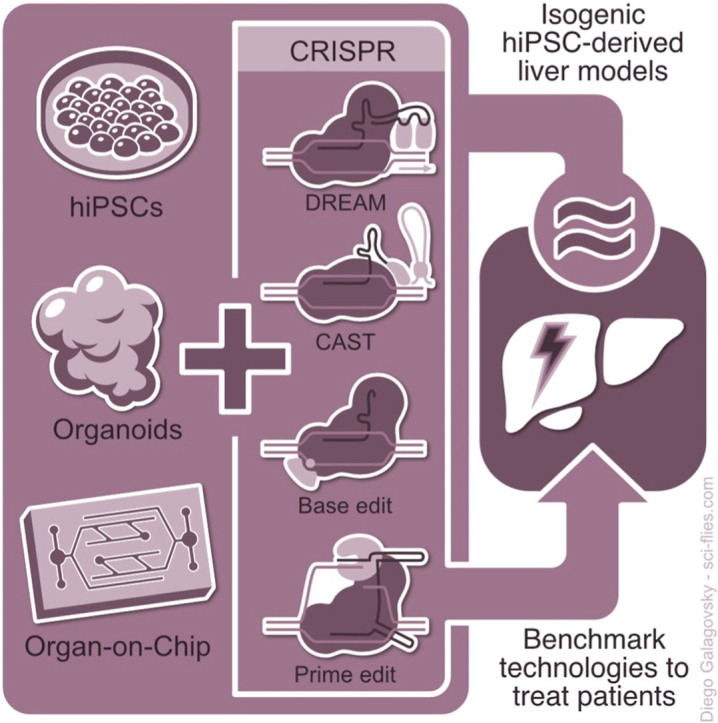

## 1 Introduction

Metabolic dysfunction-associated steatotic liver disease (MASLD), previously termed non-alcoholic fatty liver disease (NAFLD), is defined as steatotic liver disease occurring in the presence of one or more cardiometabolic risk factors and in the absence of excessive alcohol consumption [1]. MASLD encompasses a disease spectrum ranging from simple hepatic steatosis to metabolic dysfunction–associated steatohepatitis (MASH; formerly NASH), progressive fibrosis, cirrhosis, and ultimately MASH-associated hepatocellular carcinoma (HCC). The initiation and progression of MASLD are driven by a complex interplay of genetic predisposition, host metabolic status, and environmental influences [2,3] (see Table 1)

**Table 1:** Comparison of human hepatic platforms used for genome-editing–based MASLD modelling, highlighting key features, experimental utility, disease-relevant applications, and major limitations.

Platform	Key Feature	Experimental Advantage	MASLD-Relevant Application	Primary Limitation	Reference
2D hepatic culture systems	Flexible monolayer approach, Low cost	Expandable and affordable, can be used for large pharmaceutical screens	Suitable to study mitochondrial disorder in PNPLA3 variants	Absence of cellular polarity, rapid de-differentiation	([4], [5], [6])
				3D and immune micro-environment missing	
3D static liver organoids	Can mimic complex liver interactions	Long-term co-culture and passaging, prolongs, hepatic phenotype	Assess the functional impact of the GCKR variant and its relevance for stratification of patients with MASLD.	Lack of a functional vasculature, Donor-dependent variation, Functionally immature	([7], [8], [9])
3D hepatic co-culture models (primary cells)	Possibility to incorporate non-parenchy mal and stromal cells	Capture the multicellular interaction in the MASLD spectrum	Assess early MASLD fibrogenesis in *PNPLA3* variants, with optional	Donor-dependent variation, Laborious	([10], [11], [12])
			LNP-mediated gene silencing.		
Liver-on-a-chip platforms	Recapitulate key mechanical stimuli, integration of the vasculature	Immune cell perfusion models MASH recovery, study of multi-organ crosstalk feasible	Suitable for testing MASLD therapeutic drugs, capture cardio-metabolic crosstalk	Technical complexity, High cost	([13], [14], [15], [16])
Genome-edited hiPSC-derived hepatic models	Isogenic background, possibility of integrating variant,	HLA matching, ability to capture native T-cells for immune mediated studies	Correction of the CRISPR/Cas-mediat ed (Myosin Vb) MYO5B, TMSF6, A1ATDR/R variant	Time consuming and expensive to set up, On- and off target effects possible	([17], [18], [19], [20])
				Immature phenotype	

At the mechanistic level, MASH progression is characterized by a self-reinforcing cycle of hepatocellular injury and inflammation [21]. Activated macrophages release pro-inflammatory cytokines that stimulate innate immune pathways and promote the transdifferentiation of hepatic stellate cells (HSCs) into proliferative myofibroblasts, ultimately driving fibrogenesis and tissue remodeling [21]. These processes are further modulated by systemic metabolic dysfunction, including insulin resistance, hormonal imbalance, and alterations in gut microbiota, which differentially affect hepatic cell populations depending on disease stage and injury context [21,22].

The global burden of MASLD has increased substantially over the past two decades, largely driven by changes in dietary patterns, reduced physical activity, and rapid urbanization. A recent meta-analysis estimated a worldwide prevalence of 38%, representing an approximately 50% increase over 20 years, with regional variation across South America (∼30%), Asia (28–32%), and Africa (∼20%) [23,24] MASLD is now a leading cause of end-stage liver disease and liver transplantation in Europe and the United States and is associated with significant extrahepatic complications, including cardiovascular disease, type 2 diabetes, chronic kidney disease, and certain cancers [24,25]^.^

Given this growing disease burden, considerable effort has been directed toward identifying the genetic determinants of MASLD. Multi-tissue approaches, including quantitative trait locus (QTL) analysis integrated with transcriptomic, proteomic, genomic, and metabolomic datasets, have enabled the identification of susceptibility genes and associated single nucleotide polymorphisms [[26], [27], [28]]. The application of artificial intelligence-based analytical frameworks has further accelerated the discovery of candidate variants linked to complex metabolic phenotypes.

Despite these advances, a major limitation remains in understanding how these susceptibility loci mechanistically contribute to disease pathogenesis. This gap largely reflects the lack of experimental systems capable of functionally validating genetic variants in a context that recapitulates the cellular and genetic complexity of human liver disease.

Current in vivo models and conventional 3D culture systems have provided important insights into disease mechanisms; however, they exhibit significant limitations [29]. Rodent models often fail to reproduce patient-specific phenotypes and do not adequately capture human genetic diversity, while many in vitro systems lack the multicellular architecture required to model intercellular crosstalk. Furthermore, key aspects of liver pathology are species-specific, limiting the translational relevance of animal models [29,30].

These challenges are compounded by the marked genetic and clinical heterogeneity observed in MASLD, which complicates patient stratification, biomarker development, and therapeutic design. The interplay between genetic variation, metabolic dysfunction, and environmental factors leads to cell type–specific effects across hepatocytes, immune cells, and stromal populations, underscoring the need for experimental systems that can dissect these interactions with high resolution.

Human-relevant approaches, particularly CRISPR-based genome editing combined with human induced pluripotent stem cell (hiPSC) technology, provide a powerful platform to address these limitations [31]. Genome editing enables precise manipulation of disease-associated variants within isogenic backgrounds, allowing direct assessment of causal relationships. In parallel, hiPSC-derived hepatic lineages permit the generation of multiple liver cell types from defined genetic backgrounds, enabling the study of cell type–specific contributions to disease [31,32]^.^

Such systems offer the potential to bridge the gap between genetic association and functional validation. By generating isogenic hiPSC-derived hepatocyte-like cells in which risk alleles are introduced or corrected, it becomes possible to directly interrogate their effects on hepatocellular lipid metabolism and downstream pathological processes. [33]^.^ Integration of these models with organoid and microphysiological systems further enhances their capacity to recapitulate multicellular interactions and tissue-level complexity.

As the field advances toward increasingly sophisticated in vitro platforms that integrate genome editing, patient-specific stem cells, and engineered microenvironments, it becomes critical to systematically evaluate the strengths and limitations of each model. Different systems offer distinct capabilities for studying metabolic dysfunction, inflammation, and genetic variability, and their complementary use will be essential for advancing mechanistic understanding and therapeutic development in MASLD.

Building on the need for human-relevant, genetically tractable models, this review explores the integration of CRISPR-based genome editing with hiPSC-derived liver organoids and liver-on-chip platforms to study MASLD and MASH.

It focuses on.

1 Identifying existing unknown gene targets associated with specific variants
2 Linking genetic risk variants to functional phenotypes using advanced editing tools and multicellular, perfused hepatic systems.
3 Prioritizing key methods that may serve as focal points for therapeutic intervention
4 Addressing Key limitations, translational challenges, and future applications in mechanistic research, drug discovery, and precision medicine.

## 2 Methods

This study was conducted as literature review to synthesize current advances in genome editing and human induced pluripotent stem cell (hiPSC)-based hepatic models for MASLD. Relevant publications were identified through systematic searches of databases including PubMed, Nature reviews, Journal of hepatology, Web of Science, and Scopus using combinations of keywords such as “MASLD/MAFLD,” “hiPSC,” “liver organoids,” “liver-on-chip,” and “CRISPR genome editing.” Peer-reviewed original research articles and reviews were screened for relevance, with priority given to recent studies and those providing mechanistic or translational insights. Selected studies were critically evaluated and thematically categorized based on model systems, genetic approaches, and disease phenotypes to provide an integrated overview of the field.

For consistency, this review adopts a hierarchical nomenclature reflecting increasing biological complexity. hiPSCs represent the pluripotent starting point, which can be differentiated into hepatic lineages, including hepatocyte-like and non-parenchymal cells [34,35]. 3D liver organoids are formed via directed differentiation or self-organization of hepatic adult stem cells (ASCs) or (Human induced pluripotent stem cells) within a defined micro-environment [36]. More advanced microphysiological systems, such as liver-on-chip platforms, incorporate perfusion and biochemical cues to model liver function. [37]. These terms are used distinctly and are not interchangeable.

### 2.1 Stem cells

Stem cells are renewable and capable of differentiation into tissue-specific liver types depending on induction with appropriate growth factors and biochemical signalling cues. In contrast to mature primary human hepatocytes, which possess restricted proliferation and regeneration capacity after injury, stem cells serve as an unlimited cell source for liver bioengineering and therefore offer possible remedies for regenerative medicine and hepatic disorders [38,39].

### 2.2 Hepatic stem cells

Liver-derived stem cells consist of both fetal, reprogrammed, and adult liver stem cells. They have been used to produce functional hepatocytes, foetal liver stem cells, or hepatoblasts originating from the livers of developing foetuses. These cells demonstrate significant proliferation potential and an exceptional bipotential ability to develop into mature hepatocytes and cholangiocytes depending on signalling signals [40].

### 2.3 Human induced pluripotent stem cells (hiPSCs)

(hiPSCs) form the foundational cellular platform used in many liver disease models. hiPSCs are somatic cells reprogrammed into a pluripotent state capable of generating all hepatic and non-hepatic lineages [41]. They do not intrinsically possess liver-specific properties; rather, hepatic functionality arises only after directed differentiation or forward reprogramming into hepatic cell lineages [42]^.^

Through staged developmental protocols, hiPSC can be differentiated into hepatocyte-like cells (hiPSC-iHeps) that express enzymes and transporters involved in lipid metabolism, drug detoxification, and bile acid processing. Lineage specific differentiation workflows produce non-parenchymal liver cell types—including cholangiocytes, hepatic stellate cells, endothelial cells, and Kupffer-like macrophages—enabling recapitulation of the multicellular niche important for liver physiology and disease progression [43,44].

### 2.4 Liver organoid systems

The discipline of liver organoid research has achieved significant advancements in recent years, as these models represent a significant advance over 2D differentiation cultures due to their ability to recapitulate aspects of three-dimensional liver architecture and multicellular organisation. They could be derived from hiPSCS or ASCs and mimic the structural, genetic and functional features of the liver. The Clevers team first recapitulated the successful proliferation of bipotent cholangiocyte organoids (COs) derived from human adult stem cells (ASCs), capable of differentiating into liver organoids in vitro, which is currently useful to study late developmental trajectories recapitulated in MASH [[45], [46], [47], [48]].

By contrast, hiPSC-derived liver organoids emerge through the self-organization of multiple differentiating lineages and can generate structures composed of hepatocytes, cholangiocytes, stromal-like cells, and, in some protocols, immune or endothelial compartments [[49], [50], [51]].

These 3D systems support higher-order functions—including bile canaliculi formation, cell polarity, zonation-like metabolic patterning, and fibrogenic responses—making them particularly useful for modelling MASLD/MASH-related mechanisms [52]. However, to improve physiological relevance and address the limits of static 3D cultures, insights from organoids naturally progress to microphysiological systems that include perfusion, mechanical stimuli, and dynamic control of the microenvironment.

### 2.5 Microphysiological systems (MPS)

These platforms represent a complementary engineering-based technology designed to recreate organ-level functions through precisely controlled biophysical and biochemical microenvironments. MPS platforms incorporate microfluidic perfusion, shear stress, oxygen gradients, and dynamic nutrient exchange, allowing more physiologic recapitulation of the liver than static culture systems [53,54]. Liver-on-chip (LoC) devices constitute a liver-specific subclass of MPS and integrate hiPSC-derived hepatocytes, organoids, or co-cultures of primary hepatic cell types within perfused microfluidic channels.

They are able to mimic basic features of hepatic physiology—including metabolic zonation, real-time lipid flux, enhanced mitochondrial function, and continuous bile acid transport—while allowing long-term culture and sustained functional readouts [55,56]. When combined with gene-edited hepatic lineages or organoids, LoC systems provide a useful multiscale platform for evaluating variant-specific changes in metabolic and inflammatory pathways.

These in vitro platforms span increasing levels of complexity: hiPSCs as the pluripotent origin, differentiated hepatic lineages as functional units, organoids as structured 3D tissues, and microphysiological systems (LoC) providing physiologically relevant cues. While powerful, their full utility depends on alignment with the underlying molecular mechanisms of disease [57]. Accordingly, the next section examines the pathophysiology and key genetic drivers of MASLD/MASH to define the cellular and variant-specific processes these models aim to capture.

## 3 Pathophysiology and key genetic drivers of metabolic dysfunction–associated steatotic liver disease

Genetic susceptibility is a major determinant of the severity and progression of MASLD, with common and rare variants modulating lipid metabolism, inflammation, and fibrogenesis [58]^.^

**Fig. 1:**
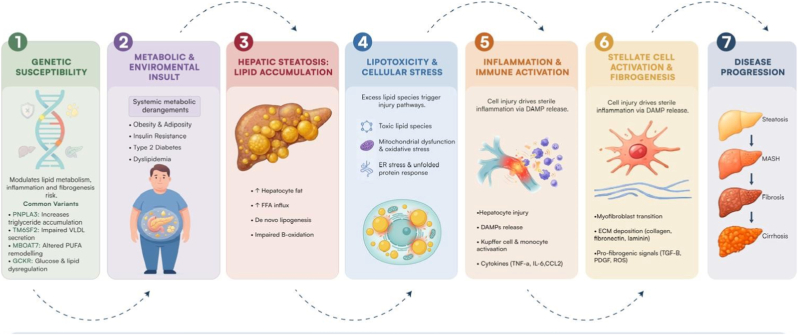
Showing Pathophysiological of MASLD from steatosis to steatohepatitis and fibrosis *Schematic overview illustrating the transition from genetic predisposition to advanced stages of metabolic dysfunction-associated steatotic liver disease (MASLD). Disease susceptibility is driven by genetic risk variants and metabolic stressors, leading to hepatic lipid accumulation(steatosis).* Progression is characterized by lipotoxicity, mitochondrial dysfunction, and activation of inflammatory pathways involving hepatocytes and resident immune cells. Subsequent crosstalk with non-parenchymal cells, including Kupffer cells, hepatic stellate cells, and endothelial cells, promotes fibrosis through extracellular matrix deposition **(Image was designed using Adobe photoshop and illustrator)**.

The pathogenesis of MASH is a complicated interplay between environmental, genetic, and metabolic factors. Hepatic steatosis, is characterised by excessive accumulation of lipids in hepatocytes usually due to increased de novo lipogenesis, decreased beta-oxidation, and increased influx of free fatty acids (FFA), central to this process [22,58,59] **(Refer to** Fig. 1**)**.

To exacerbate the process, insulin resistance increases lipotoxicity and the hepatocyte uptake of FFA. Oxidative stress, endoplasmic reticulum (ER) stress, and mitochondrial dysfunction all lead to ballooning and inflammation of hepatocytes, which in turn release damaged associated molecular patterns (DAMPS) that activate Kupffer cells, monocyte derived macrophages and hepatic stellate cells to undergo the transition to mesenchymal cells, ultimately leading to the deposition of extracellular matrix and fibrosis [59,60]. Furthermore, sterile inflammation also sits at the crossroads of metabolic injury and immune activation, further driving disease progression from lipid accumulation to irreversible hepatic damage [61].

Together, these features highlight the need to diagnose and treat MASLD/MASH with an integrated experimental strategy.

A central mechanistic feature of MASLD/MASH is lipotoxicity, characterised by the accumulation of toxic lipid species—such as saturated fatty acids, ceramides and diacylglycerols— which further induces ER stress, mitochondrial injury, altered redox balance, activation of inflammatory pathways including c-Jun N-terminal kinase, (JNK) Nuclear Factor kappa-light-chain-enhancer of activated B-cells (NF-κB), NOD-, LRR- and pyrin domain-containing protein 3 (NLRP3) inflammasome signalling [62,63]. These hepatocellular stress responses trigger the release of cytokines tumour necrosis factor (TNF-α, IL-6), chemokines (CCL2) and extracellular RNAs (eRNAs) that act as potent molecular patterns associated with danger (DAMPs). Crosstalk between hepatocytes and non-parenchymal cells eventually promotes liver sinusoidal endothelial cell dysfunction, stellate cell activation, collagen deposition, and fibrogenesis [61,64,65] **(Refer to** Fig. 1**)**.

Genome-wide association studies (GWAS), exome sequencing has provided useful mechanistic insights and found many risk alleles for MAFLD and MASH, with an estimated risk of 35 to 61% [66]. However they do not establish causal relationships, and functional interpretation often requires additional layers such as expression quantitative trait locus (eQTLs), epigenomics, CRISPR validation. Therefore, advances of in vitro platforms, with NGS-based methodologies, computational analyses and artificial intelligence can bridge this gap and position the MASLD field on the verge of transitioning from GWAS aimed at identifying prevalent genetic variations associated with disease susceptibility. Recent work has explored in detail the associated risks and underlying mechanisms of genetic susceptibility in MAFLD, encompassing lipid droplet metabolism, de novo lipogenesis, glucose metabolism, very-low-density lipoprotein (VLDL) assembly, mitochondrial dysfunction, and inflammation [67]. This subsequent section will address common genetic risk loci and with high fidelity linked with MASLD/MASH for in vitro studies application.

### 3.1 Patatin-like phospholipase domain-containing protein 3 (PNPLA3 (I148 M)

PNPLA3 functions is an important lipase regulator, enabling the hydrolysis of triacylglycerol in lipid droplets into free fatty acids. In contrast, its variants (Isoleucine-to-Methionine substitution at amino acid position) 148 M and (Serine-to-Alanine substitution at amino acid position) S47A, are the most prominent alleles associated with the MASLD/MASH population, making it one of the most important genetic drivers of disease [68]^.^ They are catalytically impaired and facilitate the accumulation of PNPLA3 and triglycerides in hepatic lipid droplets [68,69]. The variant rs738409 C > G encodes an isoleucine-to-methionine substitution at position 148, affecting the regulation of triglyceride (TG) and retinyl ester hydrolase by interacting with lipid droplets and other enzymes that modify lipids [70].

Given that the pathophysiological relevance of this regulatory pathway remains incompletely defined, the PNPLA3 I148 M variant has been consistently associated with hepatic lipid accumulation [71]. Mechanically, this variant promotes intrahepatic retention of polyunsaturated fatty acids (PUFAs) through impaired transacylation from diacylglycerol to phosphatidylcholine [72].

The I148 M variant substitution acts on lipid droplets, where it sequesters α/β-hydrolase domain–containing protein 5 (ABHD5), thereby limiting its ability to activate adipose triglyceride lipase (ATGL) and suppress triglyceride hydrolysis, this loss does not account for the steatotic phenotype; instead, the accumulation of the lipid droplets within the variant cells functionally interferes with ATGL-mediated lipolysis, resulting in a dominant gain-of-function effect that drives triglyceride accumulation [73].

These findings have implications for the potential design of refined experimental models of steatosis and MASH. Similarly to PNPLA3, the TM6SF2 variant has also been associated with hepatic steatosis and reduced plasma TG levels in humans [74].

### 3.2 Transmembrane 6 superfamily member 2 TM6SF2 (E167K)

Originally discovered in 2014, member 2 of the Transmembrane 6 superfamily (TM6SF2) is expressed in hepatocytes and intestinal epithelial cells, where it regulates hepatic lipid handling and intestinal very-low-density lipoprotein (VLDL) metabolism [75,76]. Loss-of-function variants in TM6SF2, particularly E167K, are strongly associated with metabolic dysfunction–associated steatohepatitis (MASH) promoting hepatic steatosis through impaired lipid remodeling, via interaction with PNPLA3 [77].

Deep phenotyping of additional loss-of-function variants, including leucine 156 to proline (L156P) and proline 216 to leucine (P216L), demonstrates a comparable risk of advanced steatosis and liver injury, indicating that complete loss-of TM6SF2 exacerbates disease susceptibility [78]. Experimental studies also implicate intestinal TM6SF2 deficiency in intestinal - liver axis dysfunction, characterised by altered lipid metabolism, microbiota dysbiosis, and increased endotoxin translocation, although these findings are currently limited to murine models. Together, the variants TM6SF2 and PNPLA3 illustrate convergent genetic mechanisms that link altered lipid droplet remodeling and lipoprotein secretion to hepatocellular steatosis and injury [75,78].

### 3.3 (Glucokinase regulatory protein) GCKR (P446L)

The glucokinase regulator gene (GCKR) encodes the regulatory protein glucokinase. It functions by protecting the glucose-metabolising enzyme glucokinase in hepatocytes, thus acting as a master regulator of glucose metabolism and storage in hepatic cells and pancreatic beta-cells. [79,80]^.^Wild-type GCKR regulates de novo lipogenesis (DNL) in hepatic cells by modulating glucose inflow. Two relatively frequent variants of the GCKR gene (rs1260326 and rs780094) have been linked to an increased risk of MASLD [81].

An assessment of the relationship between *GCKR* gene variants and MAFLD examined the influence of the *GCKR*rs1260326 variant on hepatic fat content, triglyceride levels, and lipoprotein concentrations in a cohort of 455 children and adolescents. The findings revealed that the *GCKR* rs1260326 variant was strongly associated with MASH and fibrosis.” [82]. On the contrary, another study showed that high liver stiffness and GCKR variation rs1260326 were associated with an increased chance of liver cirrhosis regression; regressors could be distinguished from non-regressors using a signature of circulating lipid metabolites. Subsequently, in patients with compensated cirrhosis, lipid signalling can serve as a non-invasive biomarker to identify fibrosis regression [83].

### 3.4 Membrane-bound O-acyltransferase domain–containing 7, MBOAT7 (rs641738 C > T)

(MBOAT7) is a key enzyme in the Lands pathway of phospholipid remodeling in hepatocytes and Kupffer cells. It functions by incorporating arachidonic acid via the regulation of inflammatory and fibrotic signaling through eicosanoid production [84,85]. A recent meta-analysis linked the rs641738 C > T variant with the full spectrum of MASLD, fibrosis, elevated liver enzymes, and triglycerides, although consistent associations are largely confined to individuals of European ancestry [86,87].

Mechanistic studies have shown that loss of *MBOAT7* function promotes MASH and fibrosis via upregulation of the transcriptional coactivator TAZ (WWTR1). Conversely, hepatocyte-specific restoration of *MBOAT7* attenuates fibrogenic signaling in murine models, suggesting a potential therapeutic strategy for individuals at increased risk due to genetic variants that reduce *MBOAT7* expression [88]. These variants (GCKR, MBOAT7) results in the increase of metabolic flux, phospholipid remodeling, and inflammatory pathways in MASLD progression, which is poorly captured in reductionist systems, highlighting the need for isogenic in vitro models to dissect variant-specific effects.

### 3.5 (17-beta-Hydroxysteroid dehydrogenase type 13) HSD17B13 loss-of-function variants

Loss-of-function variants in HSD17B13 have been linked with a reduced risk of chronic MASH and confer a protective effect on multiple populations, although with ancestry-dependent variability [89]. This effect is associated with decreased retinol dehydrogenase activity, altered retinol metabolism, and attenuation of fibrotic progression, and can partially offset the deleterious effects of the PNPLA3 I148 M allele [90].

The expression of HSD17B13 appears largely restricted to hepatocytes; it is currently not known if its protective effects is as a result of the direct modulation of retinoic acid signalling or indirect paracrine mechanisms, creating the opportunity for further investigation with hepatic stromal cells [91]. An early-phase clinical evaluation of the RNA interference molecule (rapirosiran) showed safety, reduced hepatic HSD17B13 expression, improvements in steatosis and lobular inflammation, supporting further therapeutic development of in vitro models [91,92]. In particular, the early phase clinical evaluation of the RNA interference therapeutic rapirosiran demonstrated favourable results.

### 3.6 (Mitochondrial amidoxime reducing component 1) MTARC 1

MTARC 1 is a key component of the mitochondrial N- reductive system mammalian enzyme dependent on molybdenum cofactor [93]. Genome-wide associated screening has shown that (MTARC1) risk alleles are related to MASH, making it a therapeutic target for the treatment of MASH. However, the MASH-relevant molecular function of mARC1 remains elusive [94]. Missense and non-nonsense variants in MTARC1 (p.A165T, rs2642438 G > A), suggest that these variants confer protection to carriers against MASH phenotypes, with reduced risk of MASH, hepatic steatosis, elevated liver enzymes, cirrhosis, liver-related mortality [94].

A study examined the role of Mtarc1 hepatocyte in the progression of MASH employing a mouse small interference RNA (siRNA) exclusively targeting hepatocytes to assess the function of mARC1 in vivo and the therapeutic potential of Mtarc1 elimination. The findings revealed that Mtarc1 knockdown protected the hepatocytes from the evolution of MASLD in diet-induced MASH animal models with obesity by decreasing hepatic mass, serum enzymes, serum lipids and hepatic triglycerides (TG). However, this was also confined to murine models, hence constraining their translational investigations in humans [95].

In contrast to other risk alleles, protective variants such as HSD17B13 and MTARC1 emphasize the importance to understudy loss-of-function mechanisms and its therapeutic potential in modulating disease mechanisms with relevant human systems.

### 3.7 APOLIPOPROTEIN B (APOB) Truncating mutation

(APOB) is a central structural component of lipoproteins, regulating the transport of hepatic and intestinal lipids through the production of APOB100 (>500 kDa) for very-low-density lipoprotein secretion in hepatocytes and APOB48 (>240 kDa) for the assembly of chylomicron in enterocytes [96]. Consequently, APOB serves as a "backbone" for the transport of lipids throughout the body, and its function is intimately linked to the maintaining hepatic-intestinal lipid homesostasis [97].

Loss-of-function in the *APOB* gene can disrupt this balance, resulting in reduced circulating APOB-containing lipoproteins, lipid malabsorption, and an increased risk of hepatic steatosis and cirrhosis, as observed in familial hypobetalipoproteinemia. Despite this, *APOB* variants are enriched in patients with advanced MASLD and are associated with lower plasma lipid levels but increased hepatic disease progression, supporting their potential application in genetic risk stratification [98,99].

### 3.8 MICROSOMAL TRIGLYCERIDE TRANSFER PROTEIN (MTTP mutations)

(MTTP) is essential for the production of APO B–containing lipoproteins from hepatic cells and absorptive enterocytes in the intestinal tract [100]. The variant MTTP has been associated with susceptibility to MASH.

A machine learning–based analysis of the UK Biobank revealed that inactivating variants in the *MTTP* gene are important for lipid regulation and are associated with markedly elevated hepatic triglyceride levels. This was assessed by comparing 19 variant carriers with 17,994 non-carriers. Carriers exhibited a median hepatic triglyceride content of 6.9% and an increased predisposition to hepatic steatosis (11.0%) compared to non-carriers. However, the analysis was limited to individuals of self-reported European ancestry.” [101,102].

APOB and MTTP mutations explain how defective hepatic lipid export can promote steatosis despite reduced circulating lipid levels, highlighting the limitations of relying on serum biomarkers alone in vivo and the need for novel organoid systems.

Collectively, these genetic variants define key metabolic, inflammatory, and fibrogenic processes observed in the MASLD/MASH spectrum. However, genetic association alone cannot establish causality, underscoring the need to complement them with CRISPR techniques that allow mechanistically dissection of variant-specific effects within human hepatic models [103]. These techniques provide the structural, functional and cues necessary to understand how these variants interact with metabolic stressors to drive disease progression. In the next section, the basic principles of gene editing will be explained.

## 4 Techniques and basic principles of gene editing

**Fig. 2:**
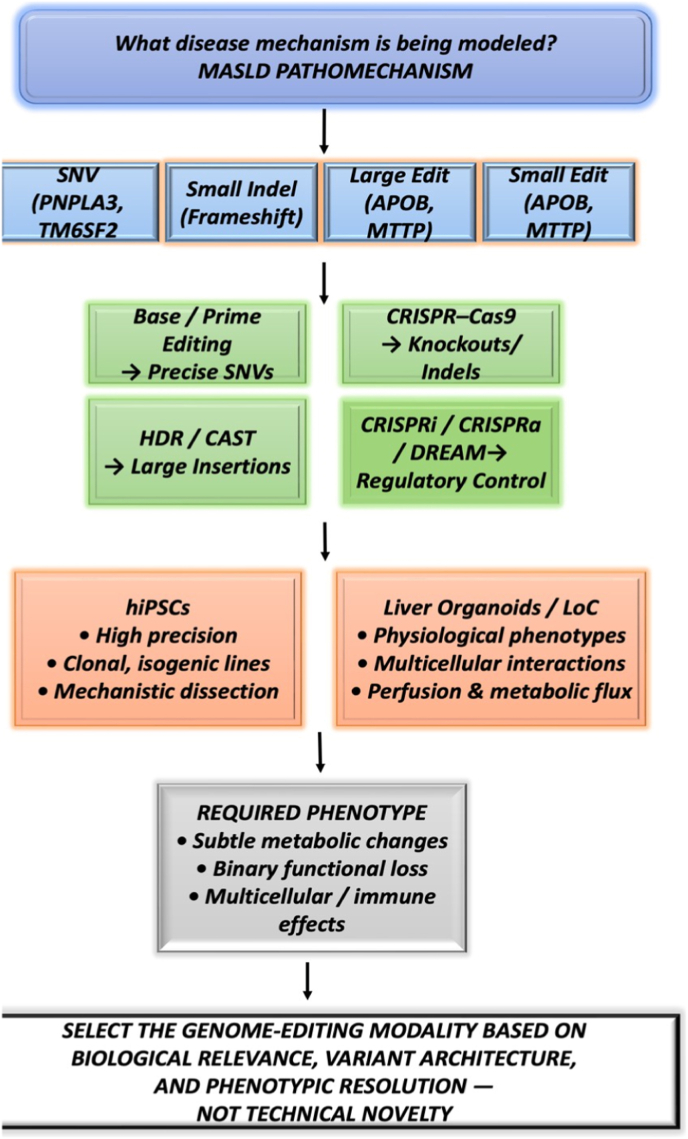
Schematic illustrating a decision tree and stepwise framework to guide the selection of the genome-editing strategy based on the hepatic disease mechanism being modelled (MASLD pathomechanism), variant architecture, editing modality, cellular context, and required phenotypic resolution. The framework emphasizes biological relevance and experimental resolution over technical novelty. **(Figure made in power-point)**.

(Variant architecture):

The genomic structure of a disease -related locus is used to pinpoint the precise variant responsible for the observed phenotype. This entails assessing the variant's position within coding or regulatory areas, its functional implications, and its association with promoters, enhancers, or splice sites [104].

(Modelling goal):

This should be aimed at recapitulating a spectrum of phenotypes observed in MASLD, offering improved quantification and insight into disease progression with relevance to human physiology [105].

(Editing strategy):

Genome-editing strategies for MASLD can be organised according to the scale and regulatory nature of the underlying variant, ranging from single-nucleotide substitutions to large structural alterations and transcriptional dysregulation **(Refer to** Fig. 2**)**.

Genome editing refers to a set of technologies that enable precise modifications to the cellular genome.“In this section,” gene editing refers to both endonuclease-based (CRISPR–Cas9-mediated double-strand break) and nuclease-independent (base and prime editing) approaches, unless otherwise specified.”

These techniques can be further classified into two main groups.

1 Specific endonuclease-based
2 Nuclease-independent platforms.

These approaches involve the introduction of DNA double-strand breaks (DSBs) by sequence-specific endonucleases, which facilitate the repair of defective genes via homologous recombination or non-homologous end joining (NHEJ) for application in liver organoids and hiPSCs [[106], [107], [108], [109]].

It can be performed in vitro or in vivo by delivering the editing machinery directly to the cells. This machinery inserts, deletes, or 'corrects' genes and induces other precise genomic alterations, providing insights into the biological role of residues or the protein itself. A range of genetic engineering strategies have been successfully applied to hiPSC- and ASC-derived organoid models, which has led to the emerging field of organoid-based genome engineering [110]. These approaches are increasingly being applied to in vitro hepatic models to enable functional investigations in the MASLD field. Consequently, current nuclease-based gene editing systems include.

### 4.1 Mega nucleases

They are widely referred to as homing endonucleases and consist of zinc finger nucleases (ZFN), transcription-activated-like effector nucleases (TALENS), and CRISPR-Cas 9 systems. Mega nucleases, ZFNs, and TALENs utilize the principle of DNA recognition domains. These methods are designed to integrate therapeutic cDNA in-frame into a 'safe harbour locus' such as the Albumin (Alb) gene, utilising its strong promoter activity in hepatic cells to ensure substantial expression of the therapeutic gene [111].

The aforementioned systems are site-specific, with the exception of CRISPR/Cas and multiplexed genome engineering, whose specificity is determined by small dual guide RNA composed of a generic transactivating cRNA and a specific CRISPR RNA. Although comprehensive progress has been achieved in the identification and engineering of disease variants, genome-wide analyses have shown that Cas9, like other programmable nucleases, can recognise and cleave DNA at off-target sites with sequences resembling on-target sites [112]^.^

This underscores the need for continued refinement of CRISPR–Cas systems through next-generation approaches that enhance on-target specificity while minimizing off-target effects, particularly in hepatic models where precision is critical for therapeutic development. In parallel, improving compatibility with widely used guide RNA variants offers additional opportunities to further increase DNA-targeting fidelity. Collectively, these advances mark a transition away from traditional double-strand break (DSB)–dependent editing toward more accurate and programmable genome-engineering strategies, along with parallel progress in delivery technologies, most notably viral delivery systems.

### 4.2 Viral delivery systems

Endonuclease-based platforms can also be combined with nuclease-independent platforms such as adeno-associated virus (AAV) and other viral or non-viral vectors, such as lipid nanoparticles (LNP). These are promising gene delivery platforms due to their low immunogenic ability to maintain persistent gene expression in non-dividing cells, such as hepatocytes, which makes them well suited for severe loss-of-function or deletion variants in MBOAT7 or HSD17B13, where long-term expression of a therapeutic transgene or protective isoform is needed, which is beneficial for MASLD research [113,114].

### 4.3 Base editors

These are created by attaching DNA deaminase enzymes to catalytically hindered Cas nucleases, allowing them to modify a single base in a specific region precisely [115]. Base editing combines the genome-targeting ability of a Cas protein with the DNA base editing function of a deaminase, which may occasionally require more regulatory elements for a more efficient function [116,117]. A study reported an in vivo base editing therapy for an infant with an inherited metabolic disorder (IMD). This ‘n-of-1’ trial involved a well-planned therapeutic pipeline, delivering a customized gene-editing treatment to an infant with neonatal-onset carbamoyl phosphate synthetase I (CPS1) deficiency within seven months after diagnosis [117]. This work demonstrated the feasibility and early success of base editing in humans for personalised therapy. This approach has the potential to treat altered metabolic disorders in hepatocytes.

Prime editing: In contrast to base editors, prime editing has been developed to fix SNPs without DSBs [118]. Prime editors (PE) are not restricted to transition changes, and hence can introduce targeted substitutions, for variants PNPLA3 I148 M or TM6SF2 E167K which require exact base changes, minor insertions, and deletions without the necessity of donor templates. Prime editing combines Cas9-nickase and reverse transcriptase with more precision. It can be used in future clinical applications to safely repair human MASLD disorders because almost no off-target effects are seen with them [118].

### 4.4 RNA editing

RNA editing, specifically antisense RNA-guided adenosine deaminase acts on RNA-mediated programmable A-to-I editing**.** This has become a potent instrument for altering RNA to facilitate the correction of pathogenic mutations and the modulation of gene expression and protein functionality [119]. The current landscape of RNA editing techniques includes small interfering RNAs (siRNAs), antisense oligonucleotides (ASOs), and emerging RNA editing technologies such as CRISPR-Cas13 [119,120]. RNA editing is suitable for variants such as (TM6SF2 or GCKR) where RNA expression can normalise transcript level modulation. According to the framework described in (Fig. 2), The successful translation of RNA editing strategies for MASLD therapeutics depends on maintaining RNA stability, achieving efficient and tissue-specific uptake and minimizing immune activation.

These criteria are most readily met in the liver because of its.

1 Pronounced trophism,
2 Highly efficient nucleic acid uptake.

In particular, these RNA-editing–based therapeutics have achieved the greatest clinical success in hepatic indications, primarily through two delivery strategies: (A) lipid nanoparticle formulations (LNP) (B) N-acetylgalactosamine (GalNAc) conjugation [121]. Importantly, these next-generation editors differ not only in precision but also in suitability, enabling the modelling of distinct variants associated with MASLD, ranging from single-nucleotide substitutions to complex transcriptomic changes.

### 4.5 CRISPR-enabled autonomous transposable systems

CAST systems, consists of.

1 Transposase subunits
2 CRISPR effectors, enable
3 RNA-guided transposition of mobile genomic elements,

They provide a method for the targeted, precise, and efficient integration of large DNA segments by reducing off-target effects. They are potentially useful for studying complex genetic disorders therefore positioning them as attractive tools in protective isoform knock in variants (HSD17B13 and GCKR)^,^ since it involves large DNA insertion rather than augmentation of a single nucleotide [122]. However, significant obstacles remain to improve efficiency and specificity, especially for medicinal applications. Current efforts seek to advance CAST systems for accurate, large-scale genome editing in liver cells [123,124].

### 4.6 CRISPR dCas9 recruited enhanced activation module (DREAM)

The CRISPR DREAM techniques use synthetic transactivation modules, which increases the scope of synthetic control over endogenous transcriptional activation. (**Refer to** Fig. 2**)**. These features are beneficial for primary cells, in vivo applications, and regulatory variants such as (HSDB17B13) that affect gene expression rather than DNA sequence integrity, where multiplexed transcription of endogenous genes or pathways is sought, providing a useful addition to the expanding toolbox [125].

### 4.7 Prime-editing-assisted site-specific integrase gene editing (PASSIGE)

Prime-editing-assisted site-specific integrase gene editing (PASSIGE) combines the programmability of prime editing with recombinases' capacity to precisely integrate huge DNA recombinases larger than 10 kilobases [126]. This capability is further enhanced through phage-assisted continuous evolution, which improves integrase efficiency and specificity useful for LOF variants such as (MBOAT7, HSD17B13) that are best addressed through targeted gene addition [126].PASSIGE can be modified to provide a Chimeric Antigen Receptor-Macrophage, an approach with broad applicability in fibrosis research for safer use. These modified cells are capable of secreting cytokines that recruit other cells to the hepatocellular tumour micro-environment [127,128].

A recent study evaluated the therapeutic efficacy of tenascin-C–targeting CAR macrophages (TNC-CAR-M) in attenuating hepatic fibrosis and elucidated the underlying antifibrotic mechanisms in vivo. The results demonstrated that TNC-CAR-M markedly reduced fibrotic progression and showed potential to restore hepatic function. However, studies were restricted to mice models, suggesting that CAR-M–based therapies may represent a promising treatment strategy for patients with MASLD in humans [128]^.^

### 4.8 Retroelement-based editing

This method is designed to facilitate the programmed integration of extensive DNA sequences with potentially enhanced efficiency and specificity. Similarly to PEs, retroelements facilitate DNA insertion through target-primed reverse transcription (TPRT), a process that involves nicking the target DNA and using the exposed 3′ end of the nick to initiate reverse transcription of retrotransposon RNA [129]. Following a second nick by restriction-like endonuclease (RLE domain), second-strand synthesis begins when the 5′ template homology binds to exposed DNA13. Cellular ligases are likely responsible for the resolution of the remaining nicks. Site-specific retrotransposons can be used as programmable genome-editing for PNPLA3 and MBOAT loss of function variant [130].

### 4.9 Artificial intelligence-based algorithms in CRISPR design and optimization

Despite all the potentials of the aforementioned methods, differences in editing results between cell types and inadvertent off-target effects continue to pose significant challenges for MASLD research. The answers to these issues can be answered by artificial intelligence (AI), which has advanced rapidly. AI is known to increase editing efficiency, predict off-target behaviours, and improve guide RNA design by utilising vast datasets from various research. Furthermore, AI could help find and create new CRISPR systems that go beyond current limitations [131]. These advances contribute to efficiency, accuracy, and safety while offering new modalities needed for the creation of customised MASLD treatments.

Importantly, as depicted in Fig. 2, the choice of genome-editing modality must align with the cellular platform—predifferentiated hepatocytes, liver organoids or liver-on-chip systems—to achieve the phenotypic resolution required to interrogate the pathogenesis of MASLD.

### 4.10 Case studies on the use of genome editing based technologies to study human metabolic liver diseases

Genome-based technologies can substantially advance our understanding of the genetic determinants underlying MASLD and support the identification of strategies for the prevention and treatment of hepatic disorders. Beyond the landmark work by Musunuru et al. [117] other studies that have provided important insights, including.

1 A proof-of-concept by Nianan et al. revealed an antifibrotic strategy in which exosome-mediated delivery of a CRISPR/dCas9-SAM system reprograms hepatic stellate cells toward a hepatocyte-like phenotype. By targeting key regulators of hepatocyte lineage (HNF4α, HGF1, and FOXA2) and enabling efficient cell-specific delivery, the approach induced phenotypic conversion in vitro and significantly reduced hepatic fibrosis in a murine CCl_4_ model, highlighting the therapeutic potential of targeted genome-based interventions in humans [132].
2 Another study by Xuexiang Han et al., developed anisamide ligand–tethered lipidoids for selective RNA delivery mediated by lipid nanoparticles for activated hepatic fibroblasts. A screening method utilized AA-lipidoids for the delivery of RNA to activated fibroblasts [133]. They showed in a preclinical hepatic fibrosis model, that targeted delivery of AA-lipolid siRNA reduced collagen deposition, and hepatic fibrosis without adverse effects [133]. Since it is crucial that hepatic fibroblast should maintain a quiescence phenotype, the integration of differentiated stellate cells from hiPSCs would be key.
3 In a similar study, a biomimetic RNA interference delivery system was developed for hepatocyte-specific targeting in non-alcoholic fatty liver disease. The integration of a tri-antennary N-acetylgalactosamine (GalNAc)-engineered cell membrane enabled efficient and safe delivery of siRNA to hepatocytes for MAFLD treatment. The platform utilizes liver-mimetic design elements to enhance uptake and specificity, facilitating efficient RNAi-mediated gene silencing in hepatocytes.” [134].
4 A clinical trial included patients with Crigler-Najjar syndrome, who lack the enzyme uridine diphosphoglucuronate glucuronosyltransferase 1A1 (UGT1A1). Crigler–Najjar causes severe unconjugated hyperbilirubinemia that requires liver transplantation [135]. This study examined the safety and efficacy of a single intravenous infusion of an adeno-associated virus serotype 8 vector, expressing UGT1A1 in the hepatocytes of patients treated with phototherapy resulting in lower unconjugated serum bilirubin. The work confirms the safety, biological activity, and therapeutic promise of AAV-mediated gene substitution for monogenic hepatic disorders informing next-generation approaches to treatments [135].

### 4.11 Clinical Comparison data

Translational relevance depends on how well in vitro models reflect clinical phenotypes and diagnostic frameworks used in MASLD. Recent large-scale analyses comparing MAFLD and MASLD—using datasets such as NHANES III and tertiary care cohorts—show that the two classifications are essentially identical, with complete overlap in patient populations. Non-invasive diagnostic tools, including (Enhanced liver fibrosis) ELF, FIB-4, and transient elastography, also use the same thresholds across MASLD, MAFLD, and NAFLD [136].

These findings support the interchangeability of clinical data and confirm that existing diagnostic algorithms remain valid despite changes in nomenclature. However, the true translational value of these clinical tools depends on their integration into biologically relevant in vitro systems, highlighting the importance of genome-edited hiPSC-derived models for accurate disease modeling.

### 4.12 Gene editing in hiPSC-derived hepatic and non-hepatic lineage cells

hiPSCs provide a scalable, isogenic platform to study disease susceptibility and progression to evaluate therapeutic candidates. Terminally differentiated hepatic cellular lineages retain donor-specific genetic backgrounds, and can be produced in unlimited quantities and are amenable to cryopreservation, making them well suited for reproducible in vitro disease modelling and high-throughput drug screening [137]. This requirement positions advanced directed hepatic differentiation protocols at the critical intersection for developing physiologically relevant, scalable disease models.

Moreover, as many hepatopathies arise from underlying genetic alterations, treatment options for advanced stages of metabolic dysfunction-associated steatotic liver disease (MASLD) are often limited to bariatric surgery or liver transplantation. In this context, combining gene-editing technologies with hiPSCs enables the correction of pathogenic variants, positioning gene editing as a promising experimental therapeutic alternative to transplantation [138,139].

This is critical, as gene editing enables precise identification of causative SNPs. However, complex linkage patterns among SNPs can complicate attribution, making it difficult to pinpoint the specific variant responsible for MASLD-associated cellular phenotypes when comparing hepatic and non-parenchymal lineages derived from disease-specific hiPSCs versus controls [140].

Furthermore, the examination of a single cell type in isolation may not comprehensively elucidate the primordial developmental trajectory and dynamic metabolic processes of a complex organ such as the liver, and employing individual cells may not adequately replicate the pathogenesis of genetic disorders impacting hepatic development and function [141]. This requires aligning gene editing approaches with liver-specific differentiation, and organoid technologies for disease modelling. [141,142]. There are important elements to consider when employing in vitro derived hepatic models for gene editing.

1 The magnitude of the mutation or SNP,
2 The age of initial disease manifestation,
3 The viability of the differentiation protocol
4 The presence of a suitable readout at the molecular or cellular level, and biochemical cues

Although next-generation editing tools offer precision in mechanism, efficiency, and capacity, their true translational value emerges only when deployed within biologically relevant systems providing the cellular context necessary to translate editing precision into disease-relevant phenotypes [142]. Therefore, the next section will highlight the advantages of current human liver organoids and LoCs platforms, how genome-editing approaches have been applied in hepatic in-vitro systems to model MASLD, evaluate therapeutic strategies, and bridge experimental findings with clinical observations.

## 5 Integrating hiPSC technology with micro-physiological systems and genome editing

**Fig. 3:**
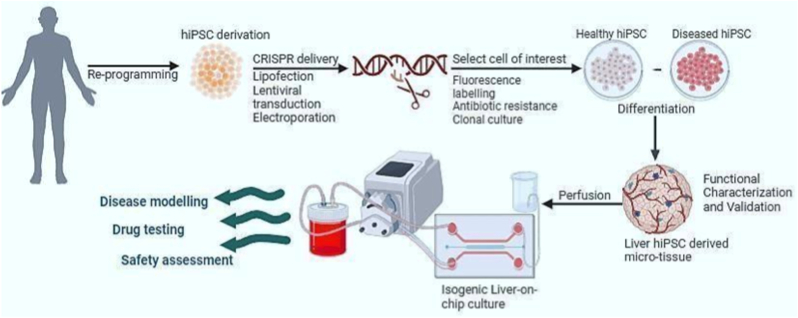
Schematic illustration of the methodology for genome engineering of human induced pluripotent stem cells (hiPSCs) into both healthy and diseased lines, combined with microphysiological systems for various applications Figure created using (Biorender.com.).

Having listed the vital elements to consider when employing human hiPSC hepatic models, the next conceptual requirement is an experimental platform capable of recapitulating not only hepatocellular lipid dysregulation, but also multicellular crosstalk, perfusion, and dynamic metabolic flux. **(Refer to** Fig. 3**)**. Microphysiological systems could prove vital in addressing this gap by providing a controlled microenvironment in which CRISPR-derived hepatic cells or organoids can be investigated under physiologically relevant conditions.

LoC models should ideally encompass all in vivo components of liver tissue, including hepatocytes, cholangiocytes, stromal cells, vasculature, and immunological elements. Significant efforts have been made to develop hepatic liver organoid (HLO) and liver-on-chip (LoC) models in recapitulating cell–cell and cell–environment interactions using hepatic cell lines, primary cells and hiPSC-based systems. These models however, still remain limited [[143], [144], [145]]. Only, few liver organoid or LoC models fully reproduce the inflammatory responses characteristic of metabolic liver diseases, especially those integrating vascular and stromal components to support resident immune cells within a 3D matrix-embedded platform.

In MASLD, Kupffer cells play a central role in modulating both pro- and anti-inflammatory signals and in regulating immune cell extravasation, making them essential for understanding immune–metabolic interactions [146]. Parenchymal cells, such as hepatocytes, along with matched immune cell populations, can now be expanded ex vivo, cryopreserved in biobanks, and potentially re-transplanted into patients to support tissue repair and regeneration [147]. This advances opportunities to investigate fundamental mechanisms of metabolic liver disease while also enabling the development of personalised therapeutic strategies. Importantly, genome-engineered cells can retain the phenotypic characteristics associated with patient-specific disease-causing mutations, preserving disease-relevant biology for study and therapeutic application [147].

This is where the intersection of hepatic-specific differentiation, organoid technology, organ-on-chip methodologies, and genome editing is relevant **(Refer to** Fig. 3**)**. Several disease-associated genes and SNPs identified through GWAS or rare genetic variants provides the opportunity to be tested in isogenic human hepatic models, where variant-inducing factors can be introduced or mitigated using suitable biochemical signals [148].

Moreover, (LoC) platforms can be further leveraged to evaluate and mitigate both on-target and off-target effects of gene editing across human tissues, thereby refining these methodologies [149]. Mechanistic disease modeling, in this context, provides a strong rationale for adopting isogenic LoC systems in preclinical drug development and testing. By combining patient-specific cells with gene editing technologies such as CRISPR–Cas, these platforms enable precise interrogation of how defined genetic alterations influence organ function and modulate responses to candidate therapeutics. The evaluation of these isogenic LoC models is based on their ability to provide innovative insights into the pathogenesis and pharmacological mechanisms of action (see Fig. 3) [150,151]. LoCs can be specifically tailored to meet the demands of the drug pipeline; They could further complement animal models (Refer to Fig. 3). Therefore, the validation of these LoCs may facilitate their initial practical use in mechanistic studies.

The initial application of LoCs for mechanistic investigations will be more practical in terms of throughput capacity, as they can help facilitate the preliminary screening of compound libraries for MASLD research. LOC systems currently incorporate.

1 Electrical,
2 Optical,
3 Mechanical
4 Biochemical sensors (that permit non-invasive on-site detection of environmental signals, cellular behaviour, and metabolic parameters) [152].

The integration of sensors into these models enables real-time evaluation of the physiological states of liver micro-environment under both healthy and diseased conditions, offering valuable indicators of metabolic secretions, hepatic function, and cellular behaviour.

Therefore, the table below outlines the applications of liver organoids and on-chip platforms for mechanistic dissection, patient stratification, and therapeutic discovery in MASLD research.

## 6 Case studies on multicellular human liver organoid (HLO) and LoC models and comparative analysis with clinical data

The application of genome editing in hepatic systems has provided important mechanistic insights into both monogenic and polygenic liver disorders relevant to MASLD. While animal models, explant cultures, and primary hepatocytes remain essential for specific questions, they lack the capacity for controlled isogenic manipulation and sustained perfusion—capabilities more effectively achieved in hepatic liver organoids (HLOs) and liver-on-chip (LoC) systems.

Building on these advantages, several studies have utilized hiPSC-derived parenchymal and non-parenchymal multicellular organoids to model key features of MASLD/MAFLD, including steatosis, lipotoxic stress, and genotype-associated risk [[153], [154], [155], [156], [157], [158]]. However, fully integrated systems incorporating all major isogenic liver cell types within a single microphysiological platform remain scarce. A consistent theme across these studies is the use of isogenic models to isolate the causal effects of genetic variants.

Recent advances in multicellular HLOs—comprising hepatocytes, Kupffer-like cells, and hepatic stellate cells—have enabled more standardized interrogation of disease phenotypes. [159]^.^ For example, miR-149-5p has been identified as a regulator of lipid metabolism and fibrosis-associated pathways, with overexpression driving lipid accumulation, inflammation, and fibrotic marker expression in organoid systems. However, such models remain limited by batch-to-batch variability, incomplete cellular maturation, and challenges in genetic manipulation, underscoring the need for further optimization [160].

In parallel, more complex perfused systems have emerged. An autologous multicellular LoC model incorporating hiPSC-derived endothelial cells and macrophages demonstrated that vascular–immune crosstalk is a key driver of fibrotic progression. Under TGFβ-1 stimulation, these systems recapitulate endothelial-to-mesenchymal transition, stellate cell activation, extracellular matrix deposition, and fibrotic marker upregulation, highlighting their relevance for modeling human fibrosis. [161]^.^ These findings establish a proof-of-concept for applying such platforms in preclinical drug testing.

Looking forward, integrating additional components such as the microbiome—combined with CRISPR-edited hiPSC models—could further enable dissection of microbiome-driven immune and metabolic regulation in MASLD.

Advances in CRISPR-based genome editing and whole-exome sequencing now allow precise engineering of hiPSCs to model disease-associated variants. This enables the generation of hepatic lineage cells carrying defined SNPs or genetic risk profiles. Notably, the *PNPLA3* I148 M variant (rs738409 G allele) is the most robustly validated, conferring increased susceptibility to MAFLD and progression to MASH. In parallel, monogenic metabolic liver disorders (in a single causal gene) have been successfully recapitulated in organoids and iHeps, underscoring their utility for personalised disease modeling. This section will therefore present key examples of gene-edited hepatic SNP models.

### 6.1 Monogenic hepatic disorders

A study demonstrated that the metabolism of deacetylase sirtuin-1 (SIRT1) is essential in the pathogenesis of MASLD. They differentiated reprogrammed hiPSCs into hepatocytes by silencing the SIRT1 gene. They found that fatty acid stimulation resulted in the downregulation of SIRT 1, and accumulation of intracellular triglycerides [162]. This proof-of-principle study outlines a method of customising liver tissue to explore the aetiology of MASLD in humans; other multifactorial elements of MASLD, including environmental influences, comprehensive metabolic profiling of liver-like cells, and additional liver-associated cell types could prove useful for future studies [163].

Takebe and colleagues produced liver organoids from patients utilising hiPSCs derived from individuals with Wolman disease, (A genetic defect in the enzyme lysosomal acid lipase). They revealed that these organoids exhibited significantly elevated levels of lipids and fibrosis compared to organoids created from healthy individuals. The findings of this study were able to recapitulate the genetic basis of Wolman disease in organoids [162]^.^ They showed similarities between the transcriptomic profiling of metabolism related genes compared to other hepatic lineages, which could be useful for translational applications.

In a follow up study, multicellular liver organoid model (HLO) with hepatocyte, stellate, and kupffer-like lineages was developed in hiPSC lines that possess a single-nucleotide variant in the glucokinase regulatory protein (GCKR) gene variant rs1260326:C > T. This study recapitulated the pathophysiology of steatohepatitis, including a fibro-inflammatory response [164].Findings revealed that inflammation cytokines, such as IL-6, TNFα, and IL-8, were released by KC-like cell-harbouring organoids.

Furthermore, they showed that a preclinical candidate TLC-065 ACMSD-specific pharmacological inhibitor, significantly reduced inhibition of mitochondrial respiratory functions, decreased activation of fibrotic pathways, improved cytoprotective and antioxidative mechanisms in GCKR treated sHLOs, similar to the therapeutic metabolic effects found in vivo mouse model of MASLD/MASH in HLO. These findings show its translational potential for individualised MASH and MASLD therapy [164].

Recent research aslo established three different syngeneic human induced pluripotent stem cell (hiPSC) lines, each possessing a single Z alpha-1 antitrypsin (ZAAT) allele, to examine the effects of ZAAT heterozygosity in hiPSC-derived hepatocytes (iHeps). The findings indicate that heterozygous MZ iHeps exhibit an intermediate pathological phenotype and exhibit similar downstream disruptions, including altered mitochondrial respiration and altered endoplasmic reticulum (ER) morphology, as observed in ZZ iHeps [165].

They also observed dysfunction in the processing of the AAT protein. However, two of the three ZZ iPSC lines examined were sourced from individuals with severe adult-onset (PiZZ1) or paediatric-onset (PiZZ6) hepatopathy, who may possess undisclosed genetic cofactors that exacerbate hepatocyte failure in the context of ZAAT expression [165].

Corrected Patient-specific induced hepatocyte-like cells (iHeps) using CRISPR-based gene editing have been shown to restore both in vitro and in vivo disease features of Wilson disease, a monogenic autosomal liver disorder. These findings suggest that gene-corrected iHeps may serve as an isogenic ex vivo cell source for potential in vivo treatment of Wilson disease and other inherited hepatopathies. However, as iHeps tend to exhibit a more fetal-like phenotype, incorporation of additional non-parenchymal cell types may be required to enhance maturation and functional fidelity [139].

Another study employed human hepatocyte organoids to mimic metabolic associated fatty liver disease (MAFLD), known as steatosis. The model used factors such as free fatty acid loading, inter-individual genetic variability (PNPLA3 I148 M) and monogenic (APOB and MTTP mutations). They utilized organoids of APOB−/− and MTTP − /− as potential targets and modulators of steatosis, employing a CRISPR screening platform [166]. Although they could replicate the multifactorial characteristics of MAFLD, complementing the model with non-parenchymal cell types in a co-culture model and the incorporation of functional vasculature could be useful for further research.

PNPLA3-associated MAFLD has been modelled using isogenic hiPSC lines carrying the I148 M variant (*PNPLA3*^I148 M^) or a deletion (*PNPLA3*^KO^) of the *PNPLA3* gene. Following forward reprogramming into hepatocytes, the cells were treated with saturated and unsaturated free fatty acids to induce MAFLD-like phenotypes, and were characterized using functional, transcriptomic, and lipidomic assays. The resulting hepatocytes exhibited increased lipid accumulation and an altered response to lipid-induced stress in *PNPLA3*^KO^ cells. They could recapitulate the early stages of steatosis, which is useful to dissect the pathophysiology of MAFLD; Other non-parenchymal cells contributing could be useful for future studies [36].

Self-organising three-dimensional hepatocyte organoids derived from human induced pluripotent stem cells have been developed to investigate the pleiotropic lipid-associated gene *TRIB1*. Using genetic perturbation and phenotypic analysis, the findings demonstrated that *TRIB1* regulates hepatocyte lipid metabolism, apolipoprotein B secretion, and intracellular triglyceride accumulation in a cell-autonomous manner [167,168]. Loss of *TRIB1* function led to increased lipid storage and impaired lipoprotein secretion, mirroring human genetic associations with dyslipidaemia and fatty liver disease [167,168]. Overall, this work establishes hiPSC-derived liver organoids as a robust platform for functional validation of GWAS loci and identifies a direct hepatocellular role for *TRIB1* in metabolic liver disease.

Although monogenic hepatic disorders provide proof-of-concept systems, MASLD is more commonly arising from polygenic risk architectures, posing additional challenges for causal modelling and patient stratification, necessitating scalable strategies.

### 6.2 Polygenic hepatic disorders

A polygenic risk score (PRS) measures an **in**dividual genetics predisposition to a disease phenotype, and patients' panels of hiPSC lines can be screened. PRS can be stratified using GWAS data and compared to control lines without polygenic risk scores or disease. Furthermore, modified polygenic risk scores for traditional clinical risk factors have been suggested to inform and educate the treatment of patients with MAFLD [169].

When formulating a PRS for MASLD several factors should be examined [170]. They include.

(1) The particular MASLD phenotype being examined
(2) Attributes of the study cohort
(3) The statistical model to be used
(4) Non genetic factors

There is a dearth of knowledge on hepatic organoids from hiPSC lines that harbour polygenic risk scores to model hepatopathies. hiPSC lines based on these scores are anticipated to create more valuable insights into the progression of MAFLD for clinical translation.

A recent study investigated the interaction of heterozygous polygenic variants in three ATP-binding cassette subfamily B member genes associated with cholestasis (*ABCB11*, *ABCB4*, and *MYO5B*) using hiPSC-derived hepatic organoids from a patient with recurrent intrahepatic cholestasis. The results showed that *MYO5B* deficiency markedly impairs canalicular transporter activity in hiPSC-derived organoids [17].

The study showed that *MYO5B*-deficient organoids exhibited reduced MRP2-mediated transport of cholyl-lysyl-fluorescein (CLF) and decreased BSEP-mediated transport of tauro-nor-THCA-24-DBD compared with control organoids. In contrast, patient-derived organoids carrying heterozygous variants in *ABCB11*, *ABCB4*, and *MYO5B*displayed a complete loss of BSEP-mediated transport while maintaining intact MRP2 function. Notably, CRISPR/Cas9 correction of the *ABCB11* variant did not restore BSEP activity, whereas correction of the *MYO5B* variant fully rescued BSEP-mediated transport. Collectively, these findings identify *MYO5B* dysfunction as the primary driver of the transport defect and highlight its potential as a target for personalised therapeutic strategies in MASLD.

When these in vitro findings are compared with clinical data, a clear translational pattern emerges clinically, MASLD exhibits near-perfect concordance, similar non-invasive fibrosis test performance (FIB-4/ELF/VCTE) (Vibration-Controlled Transient Elastography) and comparable long-term mortality rates, with modest mortality differences largely attributable to the cardiometabolic criteria embedded in the definitions of MASLD. Despite this apparent concordance between in vitro readouts and clinical endpoints, important biological aspects of MASLD—particularly immune-mediated mechanisms—are still in their infancy in current hepatic models derived from hiPSCs.

Firstly, their utility in diagnosing and evaluating genome safety strategies for patients with MASLD is still in its early stages, and as such large trials and validation are still needed to mimic the precision of these models. Secondly, because MASLD is a diverse metabolic disease with many histological manifestations, this heterogeneity affects disease progression and therapy responses. Thus, a standardised hepatic treatment model may not fully address its clinical complexity. Therefore, this only emphasizes the importance of integrating genetic screening into individualised diagnostic and treatment approaches for hiPSC-MASLD models based on the scientific question to be addressed.

Secondly, MASLD heterogeneity is strongly influenced by gut microbiota dysbiosis, with inter-individual variation in microbial composition, metabolites, and host–microbiome interactions shaping disease onset and progression [[171], [172], [173]]. Through the gut–liver axis, microbiota-derived metabolites regulate hepatic lipid metabolism, inflammation, and fibrosis, largely through innate and resident adaptive immune pathways, underscoring the need for multi-organ experimental models that better reflect clinical microbiome–hepatic interactions.

Furthermore, our understanding of alterations in lipid metabolism during hepatic zonation in MASH remains limited. Recent study utilized multizonal hepatic organoids has revealed insights into the significance of hepatic zonation, and could provide opportunities to explore liver organogenesis, metabolic balance, progression of MASLD and, ultimately, precision therapies for patients [174].

Although earlier sections highlight the general absence of an innate immune complexity, a more fundamental limitation lies in the inability to reconstruct specific lymphoid and granulocytic lineages. Furthermore, due to challenges in hiPSCs/PSC lymphoid lineage induction differentiation protocols, these hepatic MASLD models have not been used to simulate adaptive immunity, including isogenic autoreactive T and B cells and plasma cells which are particularly relevant for auto-immune-driven liver diseases such as bile sclerosing cholangitis and to understand the resolution of the inflammatory process in MASH [[175], [176], [177]].

Studies have also provided useful information on the generation of monocytes, macrophages from myeloid/erythroid progenitors, and CAR-M which can be leveraged to generate tissue-resident immune cells for MASLD therapy. Similarly, hiPSC derived innate lymphocytes like NK, γδ T, and MAIT cells remain unintegrated. Additionally, neutrophil biology — indispensable to the initiation, progression, and potential recovery phases of metabolic steatohepatitis — is under-represented in current hepatic organoid and LoC coculture models, constraining crucial mechanistic insights into innate immune contributions to MASH progression [148,[178], [179], [180]].

Beyond these biological and immunological constraints, additional technical and biophysical challenges may further limit the maturation and stability of hiPSC-derived hepatic LoC systems.

### 6.3 Technical viewpoint

Morphological traits and cellular properties are occasionally modified in a 3D static culture chip systems, particularly in traditional LoC models. Establishing 3D cell cultures is a formidable task. Moreover, due to the susceptibility of hepatocyte-like cells to shear stress, it is essential to incorporate optimal flow rate conditions that facilitate the maturation and co-culture of cell types in these LoC models, alongside simulation of biochemical factors and oxygen, as well as the elimination of cellular waste [181].

In addition, fabrication methodologies and extracellular matrix materials are essential considerations in assessing chip designs and compatibility. In addition, it is advisable to employ next-generation CRISPR technologies—DREAM, CAST, base, and prime editing— which can reduce the adverse effects on, and off-target associated with conventional CRISPR–Cas9. Consequently, these pivotal difficulties can be addressed prior to using the HLO and LoC generated by hiPSC for the development of isogenic disease liver models.

### 6.4 Translation OF IN vitro platforms and prospects

Despite significant advances in HLO and LoC technology for studying metabolic diseases in the past two decades, clinical acceptance has been sluggish due to the technological intricacies of this innovation, which originates from academic laboratories, as well as regulatory considerations concerning its integration into the medical and pharmaceutical sectors. However, with the recent approval of non-animal approaches by the National Institute of Health (NIH), much advancement in this technology is anticipated.

Secondly, a significant obstacle in pinpointing antifibrotic treatment targets in MASLD is the complexity of human liver fibrosis, which typically progresses over years or decades and encompasses various pathophysiological processes and cellular types. Contemporary single-cell or single-nucleus RNA sequencing technologies (scRNA-seq and snRNA-seq, respectively) has offered a potent new perspective to investigate human liver fibrosis with unprecedented resolution.

A combined multi-omics approach is needed to authenticate the pathogenic similarity of an in vitro model in terms of longevity to in vivo hepatic tissues at the molecular level. To date, in vitro models have only succeeded in replicating particular aspects of the in vivo disease phenotype. Industrial stakeholders must collaborate with various communities to provide standardised, resilient, repeatable, automated, and reliable platforms to bridge this gap. This does not suggest that the biological complexity and physiological significance of these models should be reduced; rather, these models must be customised to meet the different requirements of each society.

Consequently, the development of multi-organ systems that recapitulates the interplay of heredity, sex, comorbidities, and interorgan communication like the Liver-Gut, Liver-Heart axis often captured in the progression of MASLD continues to present unresolved inquiries. The selection of appropriate liver micro-environment (parenchymal cells and non-parenchymal cells) and their aggregation within a framework that facilitates cell-cell and cell-extracellular matrix interactions are crucial for biological fidelity.

Enhanced in vitro platforms are anticipated to be progressively combined with"big artificial intelligence data" to assess pharmacological molecules and identify therapeutic targets for personalised medicine strategies. Initially, precision medicine techniques are considered the ultimate solution by categorising patients according to genetic variations or disease development.

The approval of a CRISPR-based gene-editing therapy, CASGEVY, represents a significant milestone that paves the way for future uses of CRISPR therapeutics in the potential treatment of various genetic metabolic liver disorders. [182]^.^ This trial evaluated the safety and efficacy of a (CD34^+^ haematopoietic stem and progenitor cells edited by isogenic CRISPR-Cas9) in patients with severe sickle cell disease. Ex vivo CRISPR–Cas9 editing of patient-derived CD34^+^ HSPCs (targeting erythroid BCL11A enhancer), was achieved by autologous reinfusion after myeloablative conditioning [182].

This breakthrough answers important questions about how regulatory bodies assess the safety and efficacy of CRISPR technology's safety, especially for first-line therapy treatments. Although highly promising, gene-editing therapies face challenges in scaling-up, maintaining stringent safety controls, and harmonising global regulatory standards. These hurdles underscore the critical importance of foundational genome-engineering research. In parallel, patient-specific isogenic liver hiPSC models will offer a valuable platform to evaluate the efficacy and safety of emerging gene-editing therapies in a controlled and translationally relevant setting.

Furthermore, with the recent approval and the growing landscape of Resmetiron, elafibranor, FGF (19, 21) Obeticholic acid and Glucagon-Like-peptide therapy for the treatment of cardiometabolic diseases, the potential to deliver innovative test models to assess these drugs that address these unmet needs in the research of MASLD is clear. These therapies follow a classical, randomised, multi-phase clinical development pathway, with stringent efficacy endpoints, mandatory cardiovascular safety evaluation, and region-specific regulatory approvals—making them a gold standard for modern metabolic drug development [183]^.^ The increased global accessibility to effective and more economical medications is estimated to soon revolutionize the field of MASLD.

Finally, this review shows that the integration of genetic risk architecture, next generation editing tools, hiPSC technology, and microphysiological liver platforms into a unified framework is vital for modelling MASLD/MASH. These platforms will closely mimic what is observed in vivo. Therefore, by aligning variant-specific editing strategies with these perfused human hepatic systems, a translational path from genetic association to functional disease modelling is highlighted and proposed that has not been systematically addressed previously.

## Limitations of the study

Despite the significant progress achieved to date in the field of liver bioengineering as platforms for MASLD studies, substantial challenges still remain, as well as standardisation into a common framework, in achieving long-term functionality to capture the chronic aspects of MASLD.

## Declaration of competing interest

The author state no conflict of interests that may appear.

## Data Availability

No data was used for the research described in the article.

## References

[1] Li H., Yang Y., Hong W. (2020). Applications of genome editing technology in the targeted therapy of human diseases: mechanisms, advances and prospects. Sig Transduct Target Ther.

[2] European Association for the Study of the Liver (EASL); European Association for the Study of Diabetes (EASD); European Association for the Study of Obesity (EASO) (2024 Sep). EASL-EASD-EASO clinical practice Guidelines on the management of metabolic dysfunction-associated steatotic liver disease (MASLD). J. Hepatol..

[3] Rinella M.E., Lazarus J.V., Ratziu V., Francque S.M., Sanyal A.J., Kanwal F., Romero D., Abdelmalek M.F., Anstee Q.M., Arab J.P., Arrese M., Bataller R., Beuers U., Boursier J., Bugianesi E., Byrne C.D., Narro G.E.C., Chowdhury A., Cortez-Pinto H., Cryer D.R., Cusi K., El-Kassas M., Klein S., Eskridge W., Fan J., Gawrieh S., Guy C.D., Harrison S.A., Kim S.U., Koot B.G., Korenjak M., Kowdley K.V., Lacaille F., Loomba R., Mitchell-Thain R., Morgan T.R., Powell E.E., Roden M., Romero-Gómez M., Silva M., Singh S.P., Sookoian S.C., Spearman C.W., Tiniakos D., Valenti L., Vos M.B., Wong V.W., Xanthakos S., Yilmaz Y., Younossi Z., Hobbs A., Villota-Rivas M., Newsome P.N., Nafld Nomenclature (2024 Jan-Feb). A multisociety Delphi consensus statement on new nomenclature of fatty liver disease. Ann. Hepatol..

[4] Caon E., Martins M., Hodgetts H., Blanken L., Vilia M.G., Levi A., Thanapirom K., Al-Akkad W., Abu-Hanna J., Baselli G., Hall A.R., Luong T.V., Taanman J.W., Vacca M., Valenti L., Romeo S., Mazza G., Pinzani M., Rombouts K. (2024 Jun). Exploring the impact of the PNPLA3 I148M variant on primary human hepatic stellate cells using 3D extracellular matrix models. J. Hepatol..

[5] Bell C.C., Dankers A.C.A., Lauschke V.M., Sison-Young R., Jenkins R., Rowe C., Goldring C.E., Park K., Regan S.L., Walker T., Schofield C., Baze A., Foster A.J., Williams D.P., van de Ven A.W.M., Jacobs F., Houdt J.V., Lähteenmäki T., Snoeys J., Juhila S., Richert L., Ingelman-Sundberg M. (2018 Apr 1). Comparison of hepatic 2D sandwich cultures and 3D spheroids for long-term toxicity applications: a multicenter study. Toxicol. Sci..

[6] Saxton S.H., Stevens K.R. (2023 Apr). 2D and 3D liver models. J. Hepatol..

[7] Kimura M., Iguchi T., Iwasawa K., Dunn A., Thompson W.L., Yoneyama Y., Chaturvedi P., Zorn A.M., Wintzinger M., Quattrocelli M., Watanabe-Chailland M., Zhu G., Fujimoto M., Kumbaji M., Kodaka A., Gindin Y., Chung C., Myers R.P., Subramanian G.M., Hwa V., Takebe T. (2022 Oct 27). En masse organoid phenotyping informs metabolic-associated genetic susceptibility to NASH. Cell.

[8] Huang Z., Li L., Dudley K., Xiao L., Huang G., Subramaniam V.N., Chen C., Crawford R., Xiao Y. (2025 Sep 30). Three-dimensional dynamic cell models for metabolic dysfunction-associated steatotic liver disease progression. BME Front..

[9] Yuan L., Dawka S., Kim Y. (2025). Human assembloids recapitulate periportal liver tissue in vitro. Nature.

[10] Bronsard J., Savary C., Massart J., Viel R., Moutaux L., Catheline D., Rioux V., Clement B., Corlu A., Fromenty B., Ferron P.J. (2024 Feb). 3D multi-cell-type liver organoids: a new model of non-alcoholic fatty liver disease for drug safety assessments. Toxicol. Vitro.

[11] Andy Liu, A. Dylan T. Haseman, Khushal Bantu, Jia Nong, Vladimir Muzykantov, Jilian Melamed, Michael Kegel, Jenna Muscat-Rivera, Drew Weissman, David Smith, Mei Zhang, Daniel J, Rader, Tobias D. Raabe. Modeling fibrosis with MASH patient liver-derived organoids, doi:10.1101/2025.09.18.677209.

[12] Pingitore P., Sasidharan K., Ekstrand M., Prill S., Lindén D., Romeo S. (2019 Apr 2). Human multilineage 3D spheroids as a model of liver steatosis and fibrosis. Int. J. Mol. Sci..

[13] Balachander G.M., Ng I.C., Pai R.R., Mitra K., Tasnim F., Lim Y.S., Kwok R., Song Y., Yaw L.P., Quah C.B., Zhao J., Septiana W.L., Kota V.G., Teng Y., Zheng K., Xu Y., Lim S.H., Ng H.H., Yu H. (2025 Jul 8). LEADS - a comprehensive human liver-on-a-chip for non-alcoholic steatohepatitis (NASH) drug testing. Lab Chip.

[14] Xia M., Varmazyad M., Palacin I.P., Gavlock D.C., Debiasio R., LaRocca G., Reese C., Florentino R., Faccioli L.A.P., Brown J.A., Vernetti L.A., Schurdak M.E., Stern A.M., Gough A., Behari J., Soto-Gutierrez A., Taylor D.L., Miedel M. (2024 May 7). Comparison of wild-type and high-risk PNPLA3 variants in a human biomimetic liver microphysiology system for metabolic dysfunction-associated steatotic liver disease precision therapy. bioRxiv.

[15] Erin N., Tevonian, Kan Ellen L., Maniar Kairav K., Wang Alex J., Datta Anisha, Kamm Roger D., Lauffenburger Douglas A., Griffith Linda G. (2025). A vascularized liver microphysiological system captures key features of hepatic insulin resistance and monocyte infiltration. bioRxiv.

[16] Lucchetti M., Aina K.O., Grandmougin L., Jäger C., Pérez Escriva P., Letellier E., Mosig A.S., Wilmes P. (2024 Aug). An organ-on-chip platform for simulating drug metabolism along the gut-liver axis. Adv Healthc Mater.

[17] Sgodda M., Gebel E., Dignas L., Alfken S., Eggenschwiler R., Stalke A., Dröge C., Pfister E.D., Baumann U., Luedde T., Esposito I., Keitel V., Cantz T. (2025 Sep 29). iPSC-based hepatic organoids reveal a heterozygous MYO5B variant as driver of intrahepatic cholestasis. Hepatol. Commun..

[18] Wu X., Jiang D., Wang Y., Li X., Liu C., Chen Y., Sun W., He R., Yang Y., Gu X., Jiang C., Ding Q. (2024 Nov 29). Modeling metabolic-associated steatohepatitis with human pluripotent stem cell-derived liver organoids. Hepatol. Commun..

[19] Maria Morell Carola, Grace Tilson Samantha, Alexandra Tomaz Rute, Arash Shahsavari, Andi Munteanu, Giovanni Canu, Tyler Wesley Brandon, Perrin Marion, Imbisaat Geti, Mukhopadhyay Subhankar, Francesca Mazzacuva, Paul Gissen, Jose Garcia-Bernardo, Martin Bachman, Allison Rimland Casey, Fotios Sampaziotis, Irina Mohorianu, Ludovic Vallier (2024). Novel 3D approach to model non-alcoholic fatty liver disease using human pluripotent. Stem Cells eLife.

[20] Soussi F.E.A., Brusilovsky M., Buck E., Bacon W.C., Dadgar S., Fullerton A., Durban V.M., Barrile R., Helmrath M.A., Takebe T., Roth A., Kasendra M. (2025 Nov). Autologous Organoid-T cell Co-Culture platform for modeling of immune-mediated drug-induced liver injury. Adv. Sci. (Weinh.).

[21] Steinberg G.R., Carpentier A.C., Wang D. (2025 Sep 16). MASH: the nexus of metabolism, inflammation, and fibrosis. J. Clin. Investig..

[22] Sookoian S., Rotman Y., Valenti L. (2024 Nov). Genetics of Metabolic dysfunction-associated steatotic liver disease: the State of the art update. Clin. Gastroenterol. Hepatol..

[23] Feng G., Targher G., Byrne C.D., Yilmaz Y., Wai-Sun Wong V., Adithya Lesmana C.R., Adams L.A., Boursier J., Papatheodoridis G., El-Kassas M., Méndez-Sánchez N., Sookoian S., Castera L., Chan W.K., Ye F., Treeprasertsuk S., Cortez-Pinto H., Yu H.H., Kim W., Romero-Gómez M., Nakajima A., Win K.M., Kim S.U., Holleboom A.G., Sebastiani G., Ocama P., Ryan J.D., Lupşor-Platon M., Ghazinyan H., Al-Mahtab M., Hamid S., Perera N., Alswat K.A., Pan Q., Long M.T., Isakov V., Mi M., Arrese M., Sanyal A.J., Sarin S.K., Leite N.C., Valenti L., Newsome P.N., Hagström H., Petta S., Yki-Järvinen H., Schattenberg J.M., Castellanos Fernández M.I., Leclercq I.A., Aghayeva G., Elzouki A.N., Tumi A., Sharara A.I., Labidi A., Sanai F.M., Matar K., Al-Mattooq M., Akroush M.W., Benazzouz M., Debzi N., Alkhatry M., Barakat S., Al-Busafi S.A., Rwegasha J., Yang W., Adwoa A., Opio C.K., Sotoudeheian M., Wong Y.J., George J., Zheng M.H. (2024 Nov 14). Global burden of metabolic dysfunction-associated steatotic liver disease, 2010 to 2021. JHEP Rep..

[24] Ginès P., Serra-Burriel M., Kamath P.S. (2025 Jun 2). Metabolic dysfunction-associated steatotic liver disease-the new epidemic of chronic liver disease. JAMA Netw. Open.

[25] Friedman S.L. (2025 Apr 1). Fat, fibrosis, and the future: navigating the maze of MASLD/MASH. J. Clin. Investig..

[26] Zhu X.B., Hou Y.Q., Ye X.Y., Zou Y.X., Xia X.S., Yang S., Huang P., Yu R.B. (2022 May 13). Identifying and exploring the candidate susceptibility genes of cirrhosis using the multi-tissue transcriptome-wide Association Study. Front. Genet..

[27] Yetgin A. (2025 Apr 7). Revolutionizing multi-omics analysis with artificial intelligence and data processing. Quant Biol.

[28] Guadalupi G., Contini C., Iavarone F., Castagnola M., Messana I., Faa G., Onali S., Chessa L., Vitorino R., Amado F., Diaz G., Manconi B., Cabras T., Olianas A. (2023 Jul 30). Combined salivary proteome profiling and machine learning analysis provides insight into molecular signature for autoimmune liver diseases classification. Int. J. Mol. Sci..

[29] Meijnikman A.S., Fondevila M.F., Arrese M., Kisseleva T., Bataller R., Schnabl B. (2025 Apr). Towards more consistent models and consensual terminology in preclinical research for steatotic liver disease. J. Hepatol..

[30] McCarron S., Bathon B., Conlon D.M., Abbey D., Rader D.J., Gawronski K., Brown C.D., Olthoff K.M., Shaked A., Raabe T.D. (2021 Oct). Functional characterization of organoids derived from irreversibly damaged liver of patients with NASH. Hepatology.

[31] Huang Gary, Wallace Daniel F., Nathan V. (2025). Subramaniam American Journal of Physiology-Gastrointestinal and Liver Physiology.

[32] Tilson S.G., Morell C.M., Lenaerts A.S., Park S.B., Hu Z., Jenkins B., Koulman A., Liang T.J., Vallier L. (2021 Dec). Modeling PNPLA3-Associated NAFLD using human-induced pluripotent stem cells. Hepatology.

[33] Hu W., Lazar M.A. (2022 Dec). Modelling metabolic diseases and drug response using stem cells and organoids. Nat. Rev. Endocrinol..

[34] Ye L., Swingen C., Zhang J. (2013 Feb 1). Induced pluripotent stem cells and their potential for basic and clinical sciences. Curr. Cardiol. Rev..

[35] Shi Y., Deng J., Sang X., Wang Y., He F., Chen X., Xu A., Wu F. (2022 Nov 22). Generation of hepatocytes and nonparenchymal cell codifferentiation system from human-induced pluripotent stem cells. Stem Cells Int..

[36] Luce E., Messina A., Duclos-Vallée J.C. (2025 Jul-Aug). Hepatic organoids as a platform for liver disease modeling and the development of novel therapies. Clin. Res. Hepatol. Gastroenterol..

[37] Ribeiro A.J.S., Yang X., Patel V., Madabushi R., Strauss D.G. (2019 Jul). Liver microphysiological systems for predicting and evaluating drug effects. Clin. Pharmacol. Ther..

[38] Cardinale V., Lanthier N., Baptista P.M., Carpino G., Carnevale G., Orlando G., Angelico R., Manzia T.M., Schuppan D., Pinzani M., Alvaro D., Ciccocioppo R., Uygun B.E. (2023 Aug 8). Cell transplantation-based regenerative medicine in liver diseases. Stem Cell Rep..

[39] Liu Chunyan, Wang Yulian, Zhou Xuqian, Dong Lei (2024). Advances in liver engineering with cell, scaffold, and vascularization. Eng Medicine.

[40] Miyajima A., Tanaka M., Itoh T. (2014 May 1). Stem/Progenitor cells in liver development, homeostasis, regeneration, and reprogramming. Cell Stem Cell.

[41] Yao J., Yu Y., Nyberg S.L. (2022). Induced pluripotent stem cells for the treatment of liver diseases: novel concepts. Cells Tissues Organs.

[42] Rombaut M., Boeckmans J., Rodrigues R.M., van Grunsven L.A., Vanhaecke T., De Kock J. (2021 Sep). Direct reprogramming of somatic cells into induced hepatocytes: cracking the enigma code. J. Hepatol..

[43] Yu Y., Liu H., Ikeda Y., Amiot B.P., Rinaldo P., Duncan S.A., Nyberg S.L. (2012 Nov). Hepatocyte-like cells differentiated from human induced pluripotent stem cells: relevance to cellular therapies. Stem Cell Res..

[44] Starokozhko V., Hemmingsen M., Larsen L., Mohanty S., Merema M., Pimentel R.C., Wolff A., Emnéus J., Aspegren A., Groothuis G., Dufva M. (2018 May). Differentiation of human-induced pluripotent stem cell under flow conditions to mature hepatocytes for liver tissue engineering. J. Tissue Eng. Regen. Med..

[45] Osonoi S., Takebe T. (2024 May). Organoid-guided precision hepatology for metabolic liver disease. J. Hepatol..

[46] de Souza N. (2018). Organoids. Nat. Methods.

[47] Nuciforo S., Heim M.H. (2020 Oct 22). Organoids to model liver disease. JHEP Rep..

[48] Huch M., Gehart H., van Boxtel R., Hamer K., Blokzijl F., Verstegen M.M., Ellis E., van Wenum M., Fuchs S.A., de Ligt J., van de Wetering M., Sasaki N., Boers S.J., Kemperman H., de Jonge J., Ijzermans J.N., Nieuwenhuis E.E., Hoekstra R., Strom S., Vries R.R., van der Laan L.J., Cuppen E., Clevers H. (2015 Jan 15). Long-term culture of genome-stable bipotent stem cells from adult human liver. Cell.

[49] Ogawa M., Ogawa S., Bear C. (2015). Directed differentiation of cholangiocytes from human pluripotent stem cells. Nat. Biotechnol..

[50] Li Y., Nie Y., Taniguchi H. (2025 Oct 10). Protocol for generating liver organoids containing kupffer cells using human iPSCs. STAR Protoc..

[51] Zhu L., Liu S., Wang Z., Yang Y., Han P., Tong W., Zhao T., Wang L., Cui T., Yang L., Zhang Y. (2025 Apr 3). Modeling hepatic steatosis with human adult stem cell-derived liver organoids. iScience.

[52] Kim M., Yoon J., Kim D. (2025). Advanced biomanufacturing technologies for micro-physiological systems. Int. J. Precis. Eng. Manuf..

[53] Tehranirokh M., Kouzani A.Z., Francis P.S., Kanwar J.R. (2013 Oct 29). Microfluidic devices for cell cultivation and proliferation. Biomicrofluidics.

[54] Mugaanyi J., Huang J., Fang J., Musinguzi A., Lu C., Chen Z. (2025 May 22). Developments and applications of Liver-on-a-Chip technology-current status and future prospects. Biomedicines.

[55] Deguchi S., Takayama K. (2022 Dec 9). State-of-the-art liver disease research using liver-on-a-chip. Inflamm. Regen..

[56] Sharma A., Sances S., Workman M.J., Svendsen C.N. (2020 Mar 5). Multi-lineage human iPSC-Derived platforms for disease modeling and drug discovery. Cell Stem Cell.

[57] Portincasa P. (2023 Jun). NAFLD, MAFLD, and beyond: one or several acronyms for better comprehension and patient care. Intern. Emerg. Med..

[58] Gou C., Zhang W., Xu H., Zhang H., Ding R., Zhang X. (2025 Sep 13). Pathogenesis of metabolic dysfunction-associated steatotic liver disease and donor liver damage. ILIVER.

[59] Wang X., Zhang L., Dong B. (2025 Nov 1). Molecular mechanisms in MASLD/MASH-related HCC. Hepatology.

[60] Mota M., Banini B.A., Cazanave S.C., Sanyal A.J. (2016 Aug). Molecular mechanisms of lipotoxicity and glucotoxicity in nonalcoholic fatty liver disease. Metabolism.

[61] Raza S., Mahamood R., Medhe P., Shahi A., Yadav A., Tewari A., Sinha R.A. (2025 Oct 6). Sterile inflammation in MASH: emerging role of extracellular RNA and therapeutic strategies. NPJ Metab Health Dis.

[62] Bansal S.K., Bansal M.B. (2024 Jun). Pathogenesis of MASLD and MASH - role of insulin resistance and lipotoxicity. Aliment. Pharmacol. Ther..

[63] Venkatesan N., Doskey L.C., Malhi H. (2023 Dec). The role of endoplasmic reticulum in lipotoxicity during metabolic dysfunction-Associated Steatotic liver disease (MASLD) pathogenesis. Am. J. Pathol..

[64] Kostallari E., Schwabe R.F., Guillot A. (2025). Inflammation and immunity in liver homeostasis and disease: a nexus of hepatocytes, nonparenchymal cells and immune cells. Cell. Mol. Immunol..

[65] Tewari A., Rajak S., Raza S., Gupta P., Chakravarti B., Srivastava J., Chaturvedi C.P., Sinha R.A. (2023 Jul 13). Targeting extracellular RNA mitigates hepatic lipotoxicity and liver injury in NASH. Cells.

[66] Chen Y., Du X., Kuppa A., Feitosa M.F., Bielak L.F., O'Connell J.R., Musani S.K., Guo X., Kahali B., Chen V.L., Smith A.V., Ryan K.A., Eirksdottir G., Allison M.A., Bowden D.W., Budoff M.J., Carr J.J., Chen Y.I., Taylor K.D., Oliveri A., Correa A., Crudup B.F., Kardia S.L.R., Mosley T.H., Norris J.M., Terry J.G., Rotter J.I., Wagenknecht L.E., Halligan B.D., Young K.A., Hokanson J.E., Washko G.R., Gudnason V., Province M.A., Peyser P.A., Palmer N.D., Speliotes E.K. (2023 Oct). Genome-wide association meta-analysis identifies 17 loci associated with nonalcoholic fatty liver disease. Nat. Genet..

[67] Zhang X., Chang K.M., Yu J., Loomba R. (2025 Jan). Unraveling mechanisms of genetic risks in metabolic dysfunction-associated steatotic liver diseases: a pathway to precision medicine. Annu. Rev. Pathol..

[68] Johnson S.M., Bao H., McMahon C.E., Chen Y., Burr S.D., Anderson A.M., Madeyski-Bengtson K., Lindén D., Han X., Liu J. (2024 Jun 6). PNPLA3 is a triglyceride lipase that mobilizes polyunsaturated fatty acids to facilitate hepatic secretion of large-sized very low-density lipoprotein. Nat. Commun..

[69] Dong X.C. (2019 Dec 17). PNPLA3-A potential therapeutic target for personalized treatment of chronic liver disease. Front. Med..

[70] Ericson E., Bergenholm L., Andréasson A.C., Dix C.I., Knöchel J., Hansson S.F., Lee R., Schumi J., Antonsson M., Fjellström O., Nasr P., Liljeblad M., Carlsson B., Kechagias S., Lindén D., Ekstedt M. (2022 Oct). Hepatic patatin-like phospholipase domain-containing 3 levels are increased in I148M risk allele carriers and correlate with NAFLD in humans. Hepatol. Commun..

[71] Jegodzinski L., Rudolph L., Castven D., Sayk F., Rout A.K., Föh B., Hölzen L., Meyhöfer S., Schenk A., Weber S.N., Rau M., Meyhöfer S.M., Schattenberg J.M., Krawczyk M., Geier A., Mallagaray A., Günther U.L., Marquardt J.U. (2025 May 10). PNPLA3 I148M variant links to adverse metabolic traits in MASLD during fasting and feeding. JHEP Rep..

[72] Johnson S.M., Bao H., McMahon C.E., Chen Y., Burr S.D., Anderson A.M., Madeyski-Bengtson K., Lindén D., Han X., Liu J. (2024 Jun 6). PNPLA3 is a triglyceride lipase that mobilizes polyunsaturated fatty acids to facilitate hepatic secretion of large-sized very low-density lipoprotein. Nat. Commun..

[73] Wang Y., Hong S., Hudson H., Kory N., Kinch L.N., Kozlitina J., Cohen J.C., Hobbs H.H. (2025 May). PNPLA3(148M) is a gain-of-function mutation that promotes hepatic steatosis by inhibiting ATGL-Mediated triglyceride hydrolysis. J. Hepatol..

[74] Yang J., Trépo E., Nahon P., Cao Q., Moreno C., Letouzé E., Imbeaud S., Gustot T., Deviere J., Debette S., Amouyel P., Bioulac-Sage P., Calderaro J., Ganne-Carrié N., Laurent A., Blanc J.F., Guyot E., Sutton A., Ziol M., Zucman-Rossi J., Nault J.C. (2019 Feb 1). PNPLA3 and TM6SF2 variants as risk factors of hepatocellular carcinoma across various etiologies and severity of underlying liver diseases. Int. J. Cancer.

[75] Zhang X., Lau H.C.H., Ha S. (2025). Intestinal TM6SF2 protects against metabolic dysfunction-associated steatohepatitis through the gut–liver axis. Nat. Metab..

[76] Mahdessian H., Taxiarchis A., Popov S., Silveira A., Franco-Cereceda A., Hamsten A., Eriksson P., van't Hooft F. (2014 Jun 17). TM6SF2 is a regulator of liver fat metabolism influencing triglyceride secretion and hepatic lipid droplet content. Proc. Natl. Acad. Sci. U. S. A..

[77] Huang H.Y.R., Vitali C., Zhang D., Hand N.J., Phillips M.C., Creasy K.T., Scorletti E., Park J., Regeneron Centre, Schneider K.M., Rader D.J., Schneider C.V. (2024 Oct 11). Deep metabolic phenotyping of humans with protein-altering variants in TM6SF2 using a genome-first approach. JHEP Rep..

[78] Kalligeros M., Henry L.L., Younossi Z.M. (2025). Intestinal TM6SF2 and the gut-liver axis in MASLD: new insights. Metab Target Organ Damage.

[79] Kuchay M.S., Choudhary N.S., Ramos-Molina B. (2025 May 1). Pathophysiological underpinnings of metabolic dysfunction-associated steatotic liver disease. Am J Physiol Cell Physiol.

[80] Xu K., Chen P., Su Y., Chen Y., Song X., Yu B., Wang H. (2025 Oct 15). Significant association between glucokinase regulatory protein variants and genetic and metabolic diseases. Curr. Issues Mol. Biol..

[81] Husseini A.A. (2024 Jul 23). Genotypic variation in CYP2E1, GCKR, and PNPLA3 among nonalcoholic steatohepatitis patients of Turkish origin. Mol. Biol. Rep..

[82] Santoro N., Zhang C.K., Zhao H., Pakstis A.J., Kim G., Kursawe R., Dykas D.J., Bale A.E., Giannini C., Pierpont B., Shaw M.M., Groop L., Caprio S. (2012 Mar). Variant in the glucokinase regulatory protein (GCKR) gene is associated with fatty liver in obese children and adolescents. Hepatology.

[83] Mendoza Y.P., Tsouka S., Semmler G., Seubnooch P., Freiburghaus K., Mandorfer M., Bosch J., Masoodi M., Berzigotti A. (2024 Dec). Metabolic phenotyping of patients with advanced chronic liver disease for better characterization of cirrhosis regression. J. Hepatol..

[84] Meroni M., Longo M., Fracanzani A.L., Dongiovanni P. (2020 Jul). MBOAT7 down-regulation by genetic and environmental factors predisposes to MAFLD. EBioMedicine.

[85] Lee H.C., Inoue T., Imae R., Kono N., Shirae S., Matsuda S., Gengyo-Ando K., Mitani S., Arai H. (2008 Mar). Caenorhabditis elegans mboa-7, a member of the MBOAT family, is required for selective incorporation of polyunsaturated fatty acids into phosphatidylinositol. Mol. Biol. Cell.

[86] Mancina R.M., Dongiovanni P., Petta S., Pingitore P., Meroni M., Rametta R., Borén J., Montalcini T., Pujia A., Wiklund O., Hindy G., Spagnuolo R., Motta B.M., Pipitone R.M., Craxì A., Fargion S., Nobili V., Käkelä P., Kärjä V., Männistö V., Pihlajamäki J., Reilly D.F., Castro-Perez J., Kozlitina J., Valenti L., Romeo S. (2016 May). The MBOAT7-TMC4 variant rs641738 increases risk of nonalcoholic fatty liver disease in individuals of European descent. Gastroenterology.

[87] Teo K., Abeysekera K.W.M., Adams L., Aigner E., Anstee Q.M., Banales J.M., Banerjee R., Basu P., Berg T., Bhatnagar P., Buch S., Canbay A., Caprio S., Chatterjee A., Ida Chen Y.D., Chowdhury A., Daly A.K., Datz C., de Gracia Hahn D., DiStefano J.K., Dong J., Duret A., Eu-Pnafld Investigators, Emdin C., Fairey M., Gerhard G.S., Gold Consortium, Guo X., Hampe J., Hickman M., Heintz L., Hudert C., Hunter H., Kelly M., Kozlitina J., Krawczyk M., Lammert F., Langenberg C., Lavine J., Li L., Lim H.K., Loomba R., Luukkonen P.K., Melton P.E., Mori T.A., Palmer N.D., Parisinos C.A., Pillai S.G., Qayyum F., Reichert M.C., Romeo S., Rotter J.I., Im Y.R., Santoro N., Schafmayer C., Speliotes E.K., Stender S., Stickel F., Still C.D., Strnad P., Taylor K.D., Tybjærg-Hansen A., Umano G.R., Utukuri M., Valenti L., Wagenknecht L.E., Wareham N.J., Watanabe R.M., Wattacheril J., Yaghootkar H., Yki-Järvinen H., Young K.A., Mann J.P. (2021 Jan). rs641738C>T near MBOAT7 is associated with liver fat, ALT and fibrosis in NAFLD: a meta-analysis. J. Hepatol..

[88] Moore M.P., Wang X., Kennelly J.P., Shi H., Ishino Y., Kano K., Aoki J., Cherubini A., Ronzoni L., Guo X., Chalasani N.P., Khalid S., Saleheen D., Mitsche M.A., Rotter J.I., Yates K.P., Valenti L., Kono N., Tontonoz P., Tabas I. (2025 Feb 1). Low MBOAT7 expression, a genetic risk for MASH, promotes a profibrotic pathway involving hepatocyte TAZ upregulation. Hepatology.

[89] Govardhan B., Anand V.K., Nagaraja Rao P., Balachandran Menon P., Mithun S., Sasikala M., Sowmya T.R., Anuradha S., Smita C.P., Nageshwar Reddy D., Ravikanth V. (2024 Jul-Aug). 17-Beta-Hydroxysteroid dehydrogenase 13 loss of function does not confer protection to nonalcoholic fatty liver disease in Indian population. J. Clin. Exp. Hepatol..

[90] Abul-Husn N.S., Cheng X., Li A.H., Xin Y., Schurmann C., Stevis P., Liu Y., Kozlitina J., Stender S., Wood G.C., Stepanchick A.N., Still M.D., McCarthy S., O'Dushlaine C., Packer J.S., Balasubramanian S., Gosalia N., Esopi D., Kim S.Y., Mukherjee S., Lopez A.E., Fuller E.D., Penn J., Chu X., Luo J.Z., Mirshahi U.L., Carey D.J., Still C.D., Feldman M.D., Small A., Damrauer S.M., Rader D.J., Zambrowicz B., Olson W., Murphy A.J., Borecki I.B., Shuldiner A.R., Reid J.G., Overton J.D., Yancopoulos G.D., Hobbs H.H., Cohen J.C., Gottesman O., Teslovich T.M., Baras A., Mirshahi T., Gromada J., Dewey F.E. (2018 Mar 22). A protein-truncating HSD17B13 variant and protection from chronic liver disease. N. Engl. J. Med..

[91] Ma Y., Belyaeva O.V., Brown P.M., Fujita K., Valles K., Karki S., de Boer Y.S., Koh C., Chen Y., Du X., Handelman S.K., Chen V., Speliotes E.K., Nestlerode C., Thomas E., Kleiner D.E., Zmuda J.M., Sanyal A.J., Kedishvili N.Y., Liang T.J., Rotman Y., (for the Nonalcoholic Steatohepatitis Clinical Research Network) (2019 Apr). 17-Beta hydroxysteroid dehydrogenase 13 is a hepatic retinol dehydrogenase associated with histological features of nonalcoholic fatty liver disease. Hepatology.

[92] Sanyal A.J., Taubel J., Badri P., Bond S., Makarova N., Zhao W., Duggal S., Kajbaf F., Olenchock B.A., Gansner J.M. (2025 Oct). Phase I randomized double-blind study of an RNA interference therapeutic targeting HSD17B13 for metabolic dysfunction-associated steatohepatitis. J. Hepatol..

[93] Clement B., Struwe M.A. (2023 Jun 12). The history of mARC. Molecules.

[94] Emdin C.A., Haas M.E., Khera A.V., Aragam K., Chaffin M., Klarin D., Hindy G., Jiang L., Wei W.Q., Feng Q., Karjalainen J., Havulinna A., Kiiskinen T., Bick A., Ardissino D., Wilson J.G., Schunkert H., McPherson R., Watkins H., Elosua R., Bown M.J., Samani N.J., Baber U., Erdmann J., Gupta N., Danesh J., Saleheen D., Chang K.M., Vujkovic M., Voight B., Damrauer S., Lynch J., Kaplan D., Serper M., Tsao P., Million Veteran Program, Mercader J., Hanis C., Daly M., Denny J., Gabriel S., Kathiresan S. (2020 Apr 13). A missense variant in mitochondrial amidoxime Reducing Component 1 gene and protection against liver disease. PLoS Genet..

[95] Guo Y., Gao Z., LaGory E.L., Kristin L.W., Gupte J., Gong Y., Rardin M.J., Liu T., Nguyen T.T., Long J., Hsu Y.H., Murray J.K., Lade J., Jackson S., Zhang J. (2024 May 2). Liver-specific mitochondrial amidoxime-reducing component 1 (Mtarc1) knockdown protects the liver from diet-induced MASH in multiple mouse models. Hepatol. Commun..

[96] Borén J., Taskinen M.R., Packard C.J. (2024 Aug). Biosynthesis and metabolism of ApoB-Containing lipoproteins. Annu. Rev. Nutr..

[97] Lou T.W., Ren T.Y., Fan J.G. (Nov 3, 2025). Current and emerging issues in familial hypobetalipoproteinemia-related steatotic liver diseases. J. Clin. Transl. Hepatol..

[98] Di Filippo M., Moulin P., Roy P., Samson-Bouma M.E., Collardeau-Frachon S., Chebel-Dumont S., Peretti N., Dumortier J., Zoulim F., Fontanges T., Parini R., Rigoldi M., Furlan F., Mancini G., Bonnefont-Rousselot D., Bruckert E., Schmitz J., Scoazec J.Y., Charrière S., Villar-Fimbel S., Gottrand F., Dubern B., Doummar D., Joly F., Liard-Meillon M.E., Lachaux A., Sassolas A. (2014 Oct). Homozygous MTTP and APOB mutations may lead to hepatic steatosis and fibrosis despite metabolic differences in congenital hypocholesterolemia. J. Hepatol..

[99] Mureddu M., Pelusi S., Jamialahmadi O., Periti G., Ronzoni L., Heigadeh H., Malvestiti F., Saracino M., Moretti V., La Mura V., D'Ambrosio R., Petta S., Fracanzani A.L., Miele L., Vespasiani-Gentilucci U., Bugianesi E., Prati D., Schneider C.V., Romeo S., Valenti L.V.C. (2025). Carriage of rare pathogenic apob variants predispose to severe masld and hcc. Dig. Liver Dis..

[100] Raabe M., Véniant M.M., Sullivan M.A., Zlot C.H., Björkegren J., Nielsen L.B., Wong J.S., Hamilton R.L., Young S.G. (1999 May). Analysis of the role of microsomal triglyceride transfer protein in the liver of tissue-specific knockout mice. J. Clin. Investig..

[101] Tan J., Zhang J., Zhao Z., Zhang J., Dong M., Ma X., Liu S., Xin Y. (2020 Jul 23). The association between SNPs rs1800591 and rs3816873 of the MTTP gene and nonalcoholic fatty liver disease: a meta-analysis. Saudi J. Gastroenterol..

[102] Haas M.E., Pirruccello J.P., Friedman S.N., Wang M., Emdin C.A., Ajmera V.H., Simon T.G., Homburger J.R., Guo X., Budoff M., Corey K.E., Zhou A.Y., Philippakis A., Ellinor P.T., Loomba R., Batra P., Khera A.V. (2021 Dec 8). Machine learning enables new insights into genetic contributions to liver fat accumulation. Cell Genom..

[103] Joo J.H., Lee S., Kim K.P. (2025 Oct 9). Precision gene editing: the power of CRISPR-cas in modern genetics. Mol. Ther. Nucleic Acids.

[104] Lappalainen T., Li Y.I., Ramachandran S., Gusev A. (2024 Feb 29). Genetic and molecular architecture of complex traits. Cell.

[105] Hellen D.J., Ungerleider J., Tevonian E. (2026). A microphysiological model of human MASLD reveals paradoxical response to resmetirom. Commun. Biol..

[106] Benabdellah K., Sánchez-Hernández S., Aguilar-González A., Maldonado-Pérez N., Gutierrez-Guerrero A., Cortijo-Gutierrez M., Ramos-Hernández I., Tristán-Manzano M., Galindo-Moreno P., Herrera C., Martin F. (2020 Jun). Genome-edited adult stem cells: next- generation advanced therapy medicinal products. Stem Cells Transl. Med..

[107] Torella L., Santana-Gonzalez N., Zabaleta N., Gonzalez Aseguinolaza G. (2024 Oct). Gene editing in liver diseases. FEBS Lett..

[108] Stinson B.M., Loparo J.J. (2021 Jun 20). Repair of DNA double-strand breaks by the nonhomologous end joining pathway. Annu. Rev. Biochem..

[109] Belova L., Demchenko A., Erofeeva A., Kochergin-Nikitsky K., Zubkova O., Popova O., Ozharovskaia T., Salikhova D., Efremova A., Lavrov A., Smirnikhina S. (2025 Apr 4). Lung organoids from hiPSCs can be efficiently transduced by recombinant adeno-associated viral and adenoviral vectors. Biomedicines.

[110] Gaj T., Gersbach C.A., Barbas C.F. (2013 Jul). ZFN, TALEN, and CRISPR/Cas-based methods for genome engineering. Trends Biotechnol..

[111] Sharma R., Anguela X.M., Doyon Y., Wechsler T., DeKelver R.C., Sproul S., Paschon D.E., Miller J.C., Davidson R.J., Shivak D., Zhou S., Rieders J., Gregory P.D., Holmes M.C., Rebar E.J., High K.A. (2015 Oct 8). In vivo genome editing of the albumin locus as a platform for protein replacement therapy. Blood.

[112] Jinek M., Chylinski K., Fonfara I., Hauer M., Doudna J.A., Charpentier E. (2012 Aug 17). A programmable dual-RNA-guided DNA endonuclease in adaptive bacterial immunity. Science.

[113] Raigani M., Eftekhari Z., Adeli A., Kazemi-Lomedasht F. (2025 Aug 5). Advancing gene editing therapeutics: clinical trials and innovative delivery systems across diverse diseases. Mol. Ther. Nucleic Acids.

[114] Hou X., Zaks T., Langer R. (2021). Lipid nanoparticles for mRNA delivery. Nat. Rev. Mater..

[115] Anzalone A.V., Koblan L.W., Liu D.R. (2020 Jul). Genome editing with CRISPR-cas nucleases, base editors, transposases and prime editors. Nat. Biotechnol..

[116] Komor A.C., Kim Y.B., Packer M.S., Zuris J.A., Liu D.R. (2016 May 19). Programmable editing of a target base in genomic DNA without double-stranded DNA cleavage. Nature.

[117] Musunuru K., Grandinette S.A., Wang X., Hudson T.R., Briseno K., Berry A.M., Hacker J.L., Hsu A., Silverstein R.A., Hille L.T., Ogul A.N., Robinson-Garvin N.A., Small J.C., McCague S., Burke S.M., Wright C.M., Bick S., Indurthi V., Sharma S., Jepperson M., Vakulskas C.A., Collingwood M., Keogh K., Jacobi A., Sturgeon M., Brommel C., Schmaljohn E., Kurgan G., Osborne T., Zhang H., Kinney K., Rettig G., Barbosa C.J., Semple S.C., Tam Y.K., Lutz C., George L.A., Kleinstiver B.P., Liu D.R., Ng K., Kassim S.H., Giannikopoulos P., Alameh M.G., Urnov F.D., Ahrens-Nicklas R.C. (2025 Jun 12). Patient-specific In vivo gene editing to treat a rare genetic disease. N. Engl. J. Med..

[118] Schene I.F., Joore I.P., Oka R., Mokry M., van Vugt A.H.M., van Boxtel R., van der Doef H.P.J., van der Laan L.J.W., Verstegen M.M.A., van Hasselt P.M., Nieuwenhuis E.E.S., Fuchs S.A. (2020 Oct 23). Prime editing for functional repair in patient-derived disease models. Nat. Commun..

[119] Booth B.J., Nourreddine S., Katrekar D., Savva Y., Bose D., Long T.J., Huss D.J., Mali P. (2023 Jun 7). RNA editing: expanding the potential of RNA therapeutics. Mol. Ther..

[120] Freije C.A., Arechavala-Gomeza V. (2025). The current and future landscape of RNA-based therapies and diagnostics. Commun. Med..

[121] Dalabehera Manoj, Ghosh Arnab, Mohanty Satyajit, Kumar Chellappan Dinesh, Chaudhari Shubham, Ale Yogita, Poonia Neelam, Subudhi Rudra Narayan, Kaur Khanna Manmeet, Gyun Lim Hae (2025). mRNA delivery systems 2.0: engineering extrahepatic delivery for non-vaccine therapeutics. Mater. Today Bio.

[122] Klompe S.E., Jaber N., Beh L.Y., Mohabir J.T., Bernheim A., Sternberg S.H. (2022 Feb 3). Evolutionary and mechanistic diversity of type I-F CRISPR-associated transposons. Mol Cell.

[123] Tenjo-Castaño F., Rout S.S., Dey S., Montoya G. (2025 Aug). Unlocking the potential of CRISPR-associated transposons: from structural to functional insights. Trends Genet..

[124] Simoni C., Barbon E., Muro A.F., Cantore A. (2024 Aug 23). In vivo liver targeted genome editing as therapeutic approach: progresses and challenges. Front Genome.

[125] (2023 Nov). Combining compact human protein domains with CRISPR systems for robust gene activation. Nat. Methods.

[126] Pandey S., Gao X.D., Krasnow N.A. (2025). Efficient site-specific integration of large genes in mammalian cells via continuously evolved recombinases and prime editing. Nat. Biomed. Eng..

[127] Hong J., Kim Y.H. (2025 Apr 10). Cutting-edge biotherapeutics and advanced delivery strategies for the treatment of metabolic dysfunction-associated steatotic liver disease spectrum. J Control Release.

[128] Chen K.Z., Lin Z.Y., Chen L.J. (2025). TNC-targeted CAR-macrophage therapy alleviates liver fibrosis in mice. Military Med Res.

[129] Tang S., Sternberg S.H. (2023 Oct 27). Genome editing with retroelements. Science.

[130] Fell C.W., Villiger L., Lim J. (2025). Reprogramming site-specific retrotransposon activity to new DNA sites. Nature.

[131] Kim M.G., Go M.J., Kang S.H., Jeong S.H., Lim K. (2025 Jul). Revolutionizing CRISPR technology with artificial intelligence. Exp. Mol. Med..

[132] Luo N., Zhong W., Li J., Zhai Z., Lu J., Dong R. (2022 Aug). Targeted activation of HNF4α/HGF1/FOXA2 reverses hepatic fibrosis via exosome-mediated delivery of CRISPR/dCas9-SAM system. Nanomedicine (Lond).

[133] Han X., Gong N., Xue L. (2023). Ligand-tethered lipid nanoparticles for targeted RNA delivery to treat liver fibrosis. Nat. Commun..

[134] He X., Chang Z., Chen F., Zhang W., Sun M., Shi T., Liu J., Chen P., Zhang K., Guan S., Zhao Z., Li M., Dong W.F., Shao D., Yang C. (2024 Jan 15). Engineering a biomimetic system for hepatocyte-specific RNAi treatment of non-alcoholic fatty liver disease. Acta Biomater..

[135] D'Antiga L., Beuers U., Ronzitti G., Brunetti-Pierri N., Baumann U., Di Giorgio A., Aronson S., Hubert A., Romano R., Junge N., Bosma P., Bortolussi G., Muro A.F., Soumoudronga R.F., Veron P., Collaud F., Knuchel-Legendre N., Labrune P., Mingozzi F. (2023 Aug 17). Gene therapy in patients with the Crigler-Najjar syndrome. N. Engl. J. Med..

[136] Younossi Z.M., Paik J.M., Stepanova M., Ong J., Alqahtani S., Henry L. (2024 May). Clinical profiles and mortality rates are similar for metabolic dysfunction-associated steatotic liver disease and non-alcoholic fatty liver disease. J. Hepatol..

[137] Li Y., Nie Y., Taniguchi H. (2025 Oct 10). Protocol for generating liver organoids containing kupffer cells using human iPSCs. STAR Protoc..

[138] Vanmarcke G., Sai-Hong Chui J., Cooreman A., De Vos K., Cleuren L., Van Rossom R., García-Llorens G., Izuel Idoype T., Boon R., Kumar Gautam M., Castell J.V., Annaert P., Lluis F., Verfaillie C.M. (2023 Nov 5). Automated generation of hiPSC-Derived hepatic progeny by cost-efficient compounds. Stem Cell..

[139] Wei R., Yang J., Cheng C.W., Ho W.I., Li N., Hu Y., Hong X., Fu J., Yang B., Liu Y., Jiang L., Lai W.H., Au K.W., Tsang W.L., Tse Y.L., Ng K.M., Esteban M.A., Tse H.F. (2021 Oct 30;4 Oct 30). CRISPR- targeted genome editing of human induced pluripotent stem cell-derived hepatocytes for treating wilson's disease. JHEP Rep..

[140] Shi Y., Deng J., Sang X., Wang Y., He F., Chen X., Xu A., Wu F. (2022 Nov 22). Generation of hepatocytes and nonparenchymal cell codifferentiation system from Human- induced pluripotent stem cells. Stem Cells Int..

[141] Ramakrishna G., Babu P.E., Singh R., Trehanpati N. (2021 Dec). Application of CRISPR-Cas9 based gene editing to study the pathogenesis of colon and liver cancer using organoids. Hepatol. Int..

[142] Bassett A.R. (2017 Aug). Editing the genome of hiPSC with CRISPR/Cas9: disease models. Mamm. Genome.

[143] Freag M.S., Namgung B., Reyna Fernandez M.E., Gherardi E., Sengupta S., Jang H.L. (2020 Nov 29). Human nonalcoholic steatohepatitis on a chip. Hepatol. Commun..

[144] Wiriyakulsit N., Keawsomnuk P., Thongin S., Ketsawatsomkron P., Muta K. (2023 Oct 9). A model of hepatic steatosis with declined viability and function in a liver-organ-on- a-chip. Sci. Rep..

[145] Ströbel S., Kostadinova R., Fiaschetti-Egli K., Rupp J., Bieri M., Pawlowska A., Busler D., Hofstetter T., Sanchez K., Grepper S., Thoma E. (2021 Nov 23). A 3D primary human cell-based in vitro model of nonalcoholic steatohepatitis for efficacy testing of clinical drug candidates. Sci. Rep..

[146] Chen L., Guillot A., Tacke F. (2025 Jun). Reviewing the function of macrophages in liver disease. Expert Rev. Gastroenterol. Hepatol..

[147] Tomaz R.A., Zacharis E.D., Bachinger F., Wurmser A., Yamamoto D., Petrus-Reurer S., Morell C.M., Dziedzicka D., Wesley B.T., Geti I., Segeritz C.P., de Brito M.C., Chhatriwala M., Ortmann D., Saeb-Parsy K., Vallier L. (2022 Aug 12). Generation of functional hepatocytes by forward programming with nuclear receptors. eLife.

[148] Drost J., van Boxtel R., Blokzijl F., Mizutani T., Sasaki N., Sasselli V., de Ligt J., Behjati S., Grolleman J.E., van Wezel T., Nik-Zainal S., Kuiper R.P., Cuppen E., Clevers H. (2017 Oct 13). Use of CRISPR-modified human stem cell organoids to study the origin of mutational signatures in cancer. Science.

[149] Ferrari E., Visone R., Monti E., Torretta E., Moretti M., Occhetta P., Rasponi M. (2023). LivHeart: a multi organ-on-chip platform to study off-target cardiotoxicity of drugs upon liver metabolism. Adv. Mater. Technol..

[150] Mehta V., Karnam G., Madgula V. (2024 Jul 2). Liver-on-chips for drug discovery and development. Mater. Today Bio.

[151] Teng Y., Zhao Z., Tasnim F., Huang X., Yu H. (2021 Aug). A scalable and sensitive steatosis chip with long-term perfusion of in situ differentiated HepaRG organoids. Biomaterials.

[152] Asif Arun, Park Sung Hyuk, Soomro Afaque Manzoor, Khalid Muhammad Asad Ullah, Salih Abdul Rahim Chattikatikatuveli, Kang Bohye, Ahmed Faheem, Kim Kyung Hwan, Choi Kyung Hyun (2021). Microphysiological system with continuous analysisof albumin for hepatotoxicity modeling and drug screening. J. Ind. Eng. Chem..

[153] Doueiry C., Kappler C.S., Martinez-Morant C., Duncan S.A. (2024 Jul 2). A PNPLA3-Deficient iPSC-Derived hepatocyte screen identifies pathways to potentially reduce steatosis in metabolic dysfunction-associated fatty liver disease. Int. J. Mol. Sci..

[154] Tilson S.G., Morell C.M., Lenaerts A.S., Park S.B., Hu Z., Jenkins B., Koulman A., Liang T.J., Vallier L. (2021 Dec). Modeling PNPLA3-Associated NAFLD using human-induced pluripotent stem cells. Hepatology.

[155] Esteva-Font C., Maher J.J., Rulifson E., Pabst M., Bass N., Willenbring H., Mattis A.N. (2025 Nov 22). Modeling familial MASH by iPSC-Hepatocytes. medRxiv.

[156] Duwaerts C.C., Le Guillou D., Her C.L., Phillips N.J., Willenbring H., Mattis A.N., Maher J.J. (2021 Jun). Induced pluripotent stem cell-derived hepatocytes from patients with nonalcoholic fatty liver disease display a disease-specific gene expression profile. Gastroenterology.

[157] Qi L., Groeger M., Sharma A., Goswami I., Chen E., Zhong F., Ram A., Healy K., Hsiao E.C., Willenbring H., Stahl A. (2024 Sep 12). Adipocyte inflammation is the primary driver of hepatic insulin resistance in a human iPSC-based microphysiological system. Nat. Commun..

[158] Gage B.K., Liu J.C., Innes B.T., MacParland S.A., McGilvray I.D., Bader G.D., Keller G.M. (2020 Aug 6). Generation of functional liver sinusoidal endothelial cells from human pluripotent stem-cell-derived venous angioblasts. Cell Stem Cell.

[159] Li Y., Nie Y., Yang X., Liu Y., Deng X., Hayashi Y., Plummer R., Li Q., Luo N., Kasai T., Okumura T., Kamishibahara Y., Komoto T., Ohkuma T., Okamoto S., Isobe Y., Yamaguchi K., Furukawa Y., Taniguchi H. (2024 Mar 26). Integration of kupffer cells into human iPSC-derived liver organoids for modeling liver dysfunction in sepsis. Cell Rep..

[160] Correia de Sousa M., Delangre E., Berthou F., El Harane S., Maeder C., Fournier M., Krause K.H., Gjorgjieva M., Foti M. (2024 Jun 4). Hepatic miR-149-5p upregulation fosters steatosis, inflammation and fibrosis development in mice and in human liver organoids. JHEP Rep..

[161] Aina K.O. (2023). Isogenic human-induced pluripotent stem cell derived liver cells from healthy and diseased donors: a valuable tool for modeling inflammation and fibrosis. Jena.

[162] Ouchi R., Togo S., Kimura M., Shinozawa T., Koido M., Koike H., Thompson W., Karns R.A., Mayhew C.N., McGrath P.S., McCauley H.A., Zhang R.R., Lewis K., Hakozaki S., Ferguson A., Saiki N., Yoneyama Y., Takeuchi I., Mabuchi Y., Akazawa C., Yoshikawa H.Y., Wells J.M., Takebe T. (2019 Aug 6). Modeling steatohepatitis in humans with pluripotent stem cell-derived organoids. Cell Metab..

[163] Collin de l'Hortet A., Takeishi K., Guzman-Lepe J., Morita K., Achreja A., Popovic B., Wang Y., Handa K., Mittal A., Meurs N., Zhu Z., Weinberg F., Salomon M., Fox I.J., Deng C.X., Nagrath D., Soto-Gutierrez A. (2019 Aug 6). Generation of human fatty livers using Custom- engineered induced pluripotent stem cells with modifiable SIRT1 metabolism. Cell Metab..

[164] Liu Y.J., Kimura M., Li X., Sulc J., Wang Q., Rodríguez-López S., Scantlebery A.M.L., Strotjohann K., Gallart-Ayala H., Vijayakumar A., Myers R.P., Ivanisevic J., Houtkooper R.H., Subramanian G.M., Takebe T., Auwerx J. (2025 Feb). ACMSD inhibition corrects fibrosis, inflammation, and DNA damage in MASLD/MASH. J. Hepatol..

[165] Kaserman J.E., Werder R.B., Wang F., Matte T., Higgins M.I., Dodge M., Lindstrom- Vautrin Bawa P., Hinds A., Bullitt E., Caballero I.S., Shi X., Gerszten R.E., Brunetti-Pierri N., Liesa M., Villacorta-Martin C., Hollenberg A.N., Kotton D.N., Wilson A.A. (2022 Dec 6). Human iPSC-hepatocyte modeling of alpha-1 antitrypsin heterozygosity reveals metabolic dysregulation and cellular heterogeneity. Cell Rep..

[166] Hendriks D., Brouwers J.F., Hamer K., Geurts M.H., Luciana L., Massalini S., López- Iglesias C., Peters P.J., Rodríguez-Colman M.J., Chuva de Sousa Lopes S., Artegiani B., Clevers H. (2023 Nov). Engineered human hepatocyte organoids enable CRISPR-Based target discovery and drug screening for steatosis. Nat. Biotechnol..

[167] Shi Y., Deng J., Sang X., Wang Y., He F., Chen X., Xu A., Wu F. (2022 Nov 22). Generation of hepatocytes and nonparenchymal cell codifferentiation system from human-induced pluripotent stem cells. Stem Cells Int..

[168] Abbey D., Elwyn S., Hand N.J., Musunuru K., Rader D.J. (2020 Jul 8). Self-organizing human induced pluripotent stem cell hepatocyte 3D organoids inform the biology of the pleiotropic TRIB1 gene. Hepatol. Commun..

[169] Gao F., Zheng K.I., Chen S.D., Lee D.H., Wu X.X., Wang X.D., Targher G., Byrne C.D., Chen Y.P., Kim W., Zheng M.H. (2021 Mar 10). Individualized polygenic risk score identifies NASH in the eastern Asia region: a derivation and validation study. Clin. Transl. Gastroenterol..

[170] Kullo I.J., Rowley R., Dron J.S., Brockman D., Venner E., McCarthy M.I., Antoniou A.C., Easton D.F., Hegele R.A., Khera A.V., Chatterjee N., Kooperberg C., Edwards K., Vlessis K., Kinnear K., Danesh J.N., Parkinson H., Ramos E.M., Roberts M.C., Ormond K.E., Khoury M.J., Janssens A.C.J.W., Goddard K.A.B., Kraft P., MacArthur J.A.L., Inouye M., Wojcik G.L. (2021 Mar). Improving reporting standards for polygenic scores in risk prediction studies. Nature.

[171] Lonardo A., Leoni S., Alswat K.A., Fouad Y. (2020 Aug 16). History of nonalcoholic fatty liver disease. Int. J. Mol. Sci..

[172] Maher S., Rajapakse J., El-Omar E., Zekry A. (2024 Nov). Role of the gut microbiome in metabolic dysfunction-associated steatotic liver disease. Semin. Liver Dis..

[173] Jayakumar S., Loomba R. (2019 Jul). Review article: emerging role of the gut microbiome in the progression of nonalcoholic fatty liver disease and potential therapeutic implications. Aliment. Pharmacol. Ther..

[174] Reza H.A., Santangelo C., Iwasawa K. (2025). Multi-zonal liver organoids from human pluripotent stem cells. Nature.

[175] Yang Zhou J. (2023 May 2). Innate immunity and early liver inflammation. Front. Immunol..

[176] Rezvani M. (2025). Human liver immunology: from in vitro models to new insights. Cell. Mol. Immunol..

[177] Shrestha S., Jeon J.H., Hong C.W. (2025 Mar). Neutrophils in MASLD and MASH. BMB Rep..

[178] Cao X., Yakala G.K., van den Hil F.E., Cochrane A., Mummery C.L., Orlova V.V. (2019 Jun 11). Differentiation and functional comparison of monocytes and macrophages from hiPSCs with peripheral blood derivatives. Stem Cell Rep..

[179] Pierini S., Gabbasov R., Oliveira-Nunes M.C. (2025). Chimeric antigen receptor macrophages (CAR-M) sensitize HER2+ solid tumors to PD1 blockade in pre-clinical models. Nat. Commun..

[180] Ackermann M., Rafiei Hashtchin A., Manstein F., Carvalho Oliveira M., Kempf H., Zweigerdt R., Lachmann N. (2022 Feb). Continuous human iPSC-macrophage mass production by suspension culture in stirred tank bioreactors. Nat. Protoc..

[181] Leung C.M., de Haan P., Ronaldson-Bouchard K. (2022). A guide to the organ- on-a-chip. Nat. Rev. Methods Primers.

[182] Singh A., Irfan H., Fatima E., Nazir Z., Verma A., Akilimali A. (2024 May 15). Revolutionary breakthrough: FDA approves CASGEVY, the first CRISPR/Cas9 gene therapy for sickle cell disease. Ann. Med. Surg..

[183] Zheng Z., Zong Y., Ma Y. (2024). Glucagon-like peptide-1 receptor: mechanisms and advances in therapy. Sig Transduct Target Ther.

